# A diverse panel of 755 bread wheat accessions harbors untapped genetic diversity in landraces and reveals novel genetic regions conferring powdery mildew resistance

**DOI:** 10.1007/s00122-024-04582-4

**Published:** 2024-03-27

**Authors:** Rebecca Leber, Matthias Heuberger, Victoria Widrig, Esther Jung, Etienne Paux, Beat Keller, Javier Sánchez-Martín

**Affiliations:** 1 Department of Plant and Microbial Biology, University of Zurich, Zollikerstrasse 107, 8008 Zurich, Switzerland; 2 Department of Microbiology and Genetics, Spanish-Portuguese Institute for Agricultural Research (CIALE), University of Salamanca, 37007 Salamanca, Spain; 3 Université Clermont Auvergne, INRAE, GDEC, 63000 Clermont-Ferrand, France; 4 VetAgro Sup Campus Agronomique, 63370 Lempdes, France

## Abstract

**Key message:**

A bread wheat panel reveals rich genetic diversity in Turkish, Pakistani and Iranian landraces and novel resistance loci to diverse powdery mildew isolates via subsetting approaches in association studies.

**Abstract:**

Wheat breeding for disease resistance relies on the availability and use of diverse genetic resources. More than 800,000 wheat accessions are globally conserved in gene banks, but they are mostly uncharacterized for the presence of resistance genes and their potential for agriculture. Based on the selective reduction of previously assembled collections for allele mining for disease resistance, we assembled a trait-customized panel of 755 geographically diverse bread wheat accessions with a focus on landraces, called the LandracePLUS panel. Population structure analysis of this panel based on the TaBW35K SNP array revealed an increased genetic diversity compared to 632 landraces genotyped in an earlier study and 17 high-quality sequenced wheat accessions. The additional genetic diversity found here mostly originated from Turkish, Iranian and Pakistani landraces. We characterized the LandracePLUS panel for resistance to ten diverse isolates of the fungal pathogen powdery mildew. Performing genome-wide association studies and dividing the panel further by a targeted subsetting approach for accessions of distinct geographical origin, we detected several known and already cloned genes, including the *Pm2a* gene. In addition, we identified 22 putatively novel powdery mildew resistance loci that represent useful sources for resistance breeding and for research on the mildew-wheat pathosystem. Our study shows the value of assembling trait-customized collections and utilizing a diverse range of pathogen races to detect novel loci. It further highlights the importance of integrating landraces of different geographical origins into future diversity studies.

**Supplementary Information:**

The online version contains supplementary material available at 10.1007/s00122-024-04582-4.

## Introduction

Bread wheat (*Triticum aestivum* L.) provides more calories and protein per person than any other crops on Earth (FAO 2020). This allohexaploid (2*n* = 6*x* = 42, AABBDD) crop species originated from successive hybridization events, where the latest polyploidization is thought to have occurred ~ 8,000 years ago in the Fertile Crescent (Glémin et al. 2019; Haas et al. 2019). After domestication, wheat was disseminated to Europe and Asia (Bonjean and Angus 2001), where accessions were selected based on the needs of individual farmers, thus becoming locally adapted traditional accessions, so-called landraces (Zeven 1998; Villa et al. 2005). With the Green Revolution in the 1960s, landraces were systematically replaced by advanced cultivars (Evenson and Gollin 2003) at the cost of a narrower genetic diversity due to the bottleneck effect of breeding (Tanksley and McCouch 1997; Reif et al. 2005). Consequently, modern cultivars are likely to lack a substantial proportion of the genetic diversity present in the wheat gene pool to combat abiotic and biotic stresses, including infection by pathogens. Plant diseases are a big threat to agriculture, with fungal pathogens playing a major role, causing an estimated annual yield loss of 18% in wheat (Savary et al. 2019). Among those, wheat powdery mildew, caused by the obligate biotrophic ascomycete *Blumeria graminis* f.sp. *tritici* (*Bgt*), is a major source of yield loss worldwide (Savary et al. 2019). Chemical control via pesticides is expensive and can negatively impact the surrounding ecosystem (Dormann et al. 2007; Bourguet and Guillemaud 2016). Besides, the European Commission proposed binding rules to reduce EU pesticide usage by 50% until 2030 (EU 2020). Therefore, alternative strategies are needed to control wheat mildew, especially in the context of climate change, as the geographical dispersal of pathogens and the severity of their infections are expected to increase rapidly (Singh et al. 2023).

In the farm-to-fork strategy, the deployment of disease-resistant wheat accessions is proposed as a sustainable and effective way to combat pathogens (EU 2020). Such resistance can be conferred by resistance (*R*) genes, which typically encode intracellular nucleotide-binding leucine-rich repeat receptors (NLRs), albeit not always, that recognize pathogen avirulence effector proteins (AVRs) (Dodds and Rathjen 2010; Sánchez-Martín and Keller 2021; Athiyannan et al. 2022b). *R* gene resistance acts in a race-specific manner when an R protein recognizes the corresponding AVR (Flor 1971; Dodds and Rathjen 2010). The over 90 genetically characterized *R* genes against powdery mildew (*Pm* genes) represent a cornerstone of wheat breeding (McIntosh et al. 2013). However, race-specific resistance can be rapidly overcome by pathogens by evolving AVRs to evade recognition (McDonald and Linde 2002; Mundt 2014; Brown 2015). Due to this constant arms race and the low efficacy of the currently cloned *Pm* genes (Dracatos et al. 2023), the identification of new resistance genes is needed for wheat breeding programs. More than 7000 distinct NLR-encoding genes are estimated to be present in the wheat gene pool (Walkowiak et al. 2020). Based on this genomic analysis, there are possibly hundreds of potentially active but unknown *Pm* genes in the wheat germplasm. The untapped genetic diversity of wheat landraces and their adaptation to individual environments with high disease pressure of locally adapted pathogens makes them promising candidates for containing such genes (Tanksley and McCouch 1997; Zeven 1998; Müller et al. 2018).

Together with cultivars and wild relatives, landraces are conserved in gene banks, where, to date, more than 800,000 *Triticum* accessions are stored (CGIAR 2023). However, these remain mostly uncharacterized for their potential in agriculture, and their adaptive mechanisms are poorly understood, limiting their use in breeding (Tanksley and McCouch 1997; Müller et al. 2018). Attempts to unlock this hidden potential have been made using different approaches. For example, Balfourier et al. (2019) focused on maximizing the representation of different breeding statuses, dates of registration and geographical origin to reduce the collection size of the INRAe bread wheat collection from about 12,000 to 4506 accessions. Genotyping these accessions improved understanding of wheat phylogeography and genetic diversity over time. Another study characterized the genetic diversity of 80,000 accessions, which represented a large part of the CIMMYT and ICARDA germplasm banks, covering not only domesticated hexaploid wheat but also tetraploids and crop wild relatives (Sansaloni et al. 2020). This revealed unexplored diversity in landraces and wheat selection footprints. In a third recent example, Schulthess et al. (2022) genotyped the IPK winter wheat collection of 7651 accessions and a reference panel of 325 European elite cultivars. Later, this collection was phenotyped with a single powdery mildew isolate, detecting 11 previously undescribed resistance loci (Hinterberger et al. 2022).

Here, we used a panel based on former bread wheat collections assembled for allele mining (Bhullar et al. 2009, 2010b). These former collections included a main panel selected using a focused identification of germplasm strategy (FIGS) approach, revealing accessions with potentially high selection pressure for powdery mildew resistance (Mackay and Street 2004; Bhullar et al. 2009). We have now reduced these collections, focusing on landraces and maximizing the diversity of geographical origin. The reduced size of the panel allowed us to phenotype it with a diverse set of ten *Bgt* isolates, constituting a trait-customized panel that is ideal for searching for powdery mildew-resistant accessions and the underlying genes (Mascher et al. 2019). Using genome-wide association studies (GWASs) and a targeted subset approach for accessions of shared geographical origin and isolate-specific resistance patterns, we detected 22 most likely novel genetic regions associated with powdery mildew resistance.

## Materials and methods

### Plant material and growth conditions

As starting material from which we selected our working panel, we utilized a formerly assembled bread wheat collection of 1320 landraces that had been selected based on FIGS of accessions with potentially high selection pressure for powdery mildew resistance (Bhullar et al. 2009). Later, this collection was complemented with 733 accessions of diverse geographical origins (Bhullar et al. 2010b). Assessment of accession type, i.e., landrace, cultivar, breeding/research material or unknown, was based on passport data at https://www.genesys-pgr.org. When no GPS data were available for collection sites of accessions, we projected GPS using https://maps.google.com from the most detailed description available, i.e., given villages up to countries. Accessions from the two combined wheat collections were phenotyped for powdery mildew resistance at seedling stage with the six *Bgt* isolates: CHE_94202, CHE_96224, CHE_97223, CHE_97266, CHE_98230 and GBR_JIW2. The infection phenotype was used to create a reduced panel of wheat accessions, consisting of approximately 50% that showed complete resistance (0% visible infection) to one or more isolates or resistance with a threshold of 20% to at least two isolates. The remaining 50% were susceptible to all six isolates and had the same ratio of spring wheat to winter wheat as the resistant part of the panel. Additionally, we used wheat accession origin as a proxy for relatedness, choosing a geographically close susceptible counterpart for each resistant accession. The resulting diverse LandracePLUS panel of 755 bread wheat accessions (Table S1), with a focus on the fertile crescent, was infected with four additional *Bgt* isolates at seedling stage, i.e., CHE_19004, CHN_46_30, ISR_106 and ISR_94.

Differential lines used to assess virulence patterns for 27 different *Pm* genes are shown in Table S2, including near-isogenic lines (NILs) and accessions containing the designated gene. NILs had been backcrossed multiple times with susceptible accessions “Federation” or “Chancellor”, depicted by /x*Accession, where x is the number of backcrosses to the designated accession (McIntosh et al. 2013). Other differential lines were used as original seeds from the USDA ARS (https://npgsweb.ars-grin.gov/gringlobal/search) or propagated using isolation bags per single spikes.

Seeds used for infection tests were obtained by propagating accessions in the field using single rows per genotype without isolation. Seedlings for infection tests were grown in 40-well plastic trays in a growth chamber cycled at 20 °C/16 °C, 16/8 h photoperiod with 80% relative humidity.

### Powdery mildew isolates and infections

We used previously sampled and sequenced *Bgt* isolates CHE_94202, CHE_96224, CHE_97223, CHE_97266, CHE_98230, CHN_46_30, GBR_JIW2, ISR_94 and ISR_106, which are described by Sotiropolous (Sotiropoulos et al. 2022) (Table S3). We sampled chasmothecia of one additional isolate, CHE_19004 (Table S3), in 2019 from a wheat field at Reckenholz, Affoltern, Switzerland, which was revived and sequenced as previously described (Sotiropoulos et al. 2022).

Powdery mildew infection tests of the differential lines and the LandracePLUS panel accessions were carried out on the primary leaves of 10 to 15-day-old seedlings grown under the abovementioned conditions. Leaf segments were placed with their adaxial side up in Petri dishes filled with 0.5% Phyto agar containing 30 ppm benzimidazole. Fresh conidiospores were dispersed using 5-ml Pasteur glass pipettes in a settling tower (Lutz et al. 1992). Petri dishes with detached leaf segments were incubated for 7 to 9 days at 20 °C, 80% relative humidity with a 16 h light/8 h dark cycle and 50 μmol m^−2^ s^−1^ photon flux density. Infections were done in batches, with replicates of leaf segments from at least three independent seedlings per wheat accession on the same petri dish, infected at the same time. We used mildew susceptible accession Kanzler as a control for a proper mildew infection for all infection tests. Accessions Chancellor and Federation were used as additional susceptible controls for tests on differential lines. These susceptible controls were grown together with the tested accessions of each batch and distributed throughout the layout petri dish. If controls were not well infected and, in addition, the overall infection was low, the full infection test was repeated rather than using controls as a means to correct phenotypic values.

Disease levels were assessed 7 to 9 days after inoculation, depending on fungal growth in the batch, using a discrete quantitative scale with a score from 0 to 100 for the percentage of leaf area covered by sporulating mildew colonies, as described earlier (Kaur et al. 2008). Disease levels of differential lines were directly scored as resistant (< = 20) and susceptible (> 20).

### DNA extraction and genotyping

DNA extraction of plant material was performed as previously described (Stein et al. 2001). DNA quality was assessed via agarose gels and genotyped using the TaBW35K single-nucleotide polymorphism (SNP) array (Paux et al. 2022). The SNP call dataset included marker positions and flanking sequences based on RefSeq v1.0 of Chinese Spring (IWGSC 2018).

### Pm gene screening via polymerase chain reactions (PCRs) and sequencing

PCR analysis was performed using *Pm4* (Sánchez-Martín et al. 2021) and *Pm2* haplotype-specific markers (Manser et al. 2021). Four random landraces that were identified to carry *Pm2* were then used for long-range and a following nested PCR to amplify the gene for Sanger sequencing as previously described (Sánchez-Martín et al. 2016).

### General data analysis and visualization

Unless indicated otherwise, analyses were done using R version 3.6.3 (R Core Team 2022), including data handling with Tidyverse (Wickham et al. 2023) and visualizations with R package ggplot2 version 3.3.6 (Wickham 2016).

Kinship matrices for hierarchical clustering and visualization were done with GAPIT version 3 (Wang and Zhang 2021). Dendrogram formation and hierarchical clustering were performed using the stats R package version 3.6.3 (R Core Team 2022) functions hclust (method = ward.D2) and dist (method = euclidean). Defining clusters of genotypes was done using the package dendextend version 1.16.0 (Galili 2015).

Wheat *Pm* gene sequence assembly and alignment was done with CLC Genomics Workbench version 20.0.4 (Qiagen Bioinformatics, https://digitalinsights.qiagen.com/). Pathogen *Avr* gene sequence alignment was done using IGV version 2.15.4 (Robinson et al. 2011).

### SNP filtering and file format

We filtered for “PolyHighResolution” or off-target variants (OTVs) and markers with known chromosomal positions in the Chinese Spring RefSeq v1.0 reference genome. Thresholds of 25% heterozygosity, 25% missing data per wheat accession and 5% missing data per marker were applied, and markers with duplicated positions were removed. Absent haplotypes of an OTV were translated to “NA” to facilitate their inclusion in downstream analyses. Taken together, this resulted in 29,965 polymorphic markers. These were brought into a Hapmap format with R and then transformed into a variant call format (VCF) file using the software TASSEL version 5.0 (Bradbury et al. 2007). This file was used as an input to generate plink files using vcftools version 0.1.16 (Danecek et al. 2011), which were then transformed together with phenotyping data into bed, bim and fam files using PLINK v1.07 (Purcell et al. 2007) for Admixture and association analyses.

### Phenotypic data analysis

The raw median of the biological replicates was taken as the final phenotype for seedling resistance assessment. Inconclusive phenotypes, e.g., 50% resistant and 50% susceptible against the same isolate due to possible seed contamination or heterozygosity, were excluded from further analyses. Phenotypes with less than three replicates were also excluded. These values were transformed into two categories, where 0–20% = resistant and > 20% = susceptible for GWAS and Mantel tests. For Pearson’s correlation, phenotypic values of the differential lines were transformed to 0 and 1, respectively.

Pearson’s correlation between the isolate phenotypes was calculated using the stats package and visualized with the package corrplot version 0.92 (Wei and Simko 2021). Heritability was calculated for each pathogen isolate using R package lme4 version 1.1-34 (Bates et al. 2015) and a nested linear mixed model approach, where fixed variance is defined as the wheat genotype, and random variance as the infection test batch (“Round”) nested within the specific petri dish (“Plate”). *P*-values are based on ANOVA tests between the full and null models.

### In silico genotyping of TaBW35K array SNP variants in high-quality sequenced wheat genomes

For the comparison of genetic diversity, Fielder (Sato et al. 2021), Renan (Aury et al. 2022) and the 10 + wheat reference genomes ArinaLrFor, Chinese Spring, Claire, Cadenza, Jagger, Julius, Landmark, Lancer, Mace, Paragon, Norin61, Robigus, Stanley, SYMattis and Weebill (IWGSC 2018; Walkowiak et al. 2020) were added. Flanking sequences of SNP array markers were queried using BLASTN searches against the publicly available wheat genomes. Blast results were filtered for hits with at least 60 bp alignment and 96% shared identity, allowing no more than three mismatched nucleotides. Positions of the SNPs were then extracted from BLASTN alignments on the respective genomes through in-house scripts. Merging the resulting dataset with the 29,965 previously used markers resulted in an overlap of 27,337 SNPs used for principal component analysis (PCA) and hierarchical cluster analysis.

### Diversity Analysis via PCA and hierarchical clustering

PCAs for the LandracePLUS panel and high-quality sequenced genomes were done based on the 27,337 SNP set using the R package SNPRelate version 1.18.1 and gdsfmt version 1.20.0 (Zheng et al. 2012). For the PCA for the comparison to the 632 landraces from the INRAe study (Balfourier et al. 2019), we first filtered the genotyping data of the combined datasets provided by INRAe for the same cleaned 29,965 SNPs of the LandracePLUS panel. Because no creation of a Hapmap or VCF file was necessary for further downstream analysis, this dataset could be directly used for PCA using the prcomp function.

Hierarchical clustering analysis was performed using the kinship matrix of the 27,337 SNP set, including high-quality sequenced genomes. We visualized the dendrogram using dendextend version 1.16.0 (Galili 2015).

### Admixture kinship analysis and comparative visualization

We used the.bed file of 29,965 SNPs (not including the chromosome-assembled genomes) as an input to assess population structure using ADMIXTURE version 1.3.0 (Alexander et al. 2009), where the most likely number of founder populations K can be estimated via running the model over a series of values of K and then choosing K around the lowest occurring cross-validation (CV) error. We ran the model for K = 2 to K = 20. However, the CV error steadily dropped with increasing K (Fig. S1). We, therefore, regarded Ks after the largest CV error drops of 33% in total as appropriate estimations for population structure, i.e., K = 4 to K = 6. A bootstrap of 500 and CV of 10 was used for this analysis.

A kinship matrix for the visualization was made with the same 29,965 SNP set using GAPIT version 3 (Wang and Zhang 2021). The kinship matrix was used as an input for hierarchical clustering for the comparative dendrogram. This dendrogram was visualized using the R package ggdendro version 0.1.23 (de Vries and Ripley 2022). Finally, these two plots and the admixture barplot were merged using the R package patchwork version 1.1.1 (Pedersen 2020).

### Mantel test

To calculate the Mantel test, we first transformed the VCF file of 29,965 SNPs into genlight format using the R package vcfR version 1.14.0 (Knaus and Grünwald 2017). Then, we used this as an input for producing a Bray–Curtis genetic distance matrix with the vegdist function from R package vegan 2.6-4 (Oksanen et al. 2022). This genetic matrix was then correlated to the Euclidean phenotypic distance matrices using the vegan package mantel function with the Spearman method and 999 permutations to obtain Mantel r values.

### Genome-wide association analysis (GWAS)

For GWAS, missing data from the 29,965 SNP set were imputed using general Beagle version 5.4 (Browning et al. 2018). GWAS and estimates of effect size—beta—were calculated using the GEMMA (Zhou and Stephens 2012) univariate linear mixed model. For the full LandracePLUS panel and each subset, bed, bim and fam files were created as described above, fam files were used as input for creating the kinship matrix in GEMMA using the options -gk 1 and -miss 1. MAF was set to 1% for the full LandracePLUS panel and all subsets above 300 accessions in size, while for the other runs, the MAF was set to 5%. This matrix was then integrated into the univariate linear-mixed model with option -miss 1 (Zhou and Stephens 2012). Phenotypic data for the association studies were added to the fam in R. *P*-values were based on the likelihood ratio test, and -log10 transformed for Manhattan plots. We used two thresholds to account for multiple testing: the false discovery rate (FDR) and the more conservative Bonferroni correction (BC). However, we regarded SNPs that passed the FDR test as significant.

Due to the design of the TaBW35K SNP array based on linkage disequilibrium (LD), meaningful LD decay analysis was not possible. We, therefore, decided to define GWAS peak intervals based on the LD decay results of a recent study on bread wheat using over 40 million SNPs (Liu et al. 2023). There, LD decay was found to be 6.0353, 2.3851 and 3.0278 Mb for subgenomes A, B and D, respectively. Hence, we defined peaks in a subgenome-dependent manner, as regions where at least two SNPs were significantly associated with the powdery mildew phenotype within the range of the above LD decay bp distances. We further considered significant single SNP associations as peaks if they uniquely mapped to one chromosome (weak homology on homoeologs, i.e., at least six additional SNPs/gaps), were surrounded by no or few SNPs, and (1) they occurred for more than one isolate or (2) their significance passed the more stringent BC threshold. The most significant SNP—the peak SNP—plus/minus the subgenome-specific LD decay distance was used to define each peak interval. Alternatively, if several peak SNPs occurred, both were used for the interval calculation.

To test if the peak on chromosome 5D was derived from *Pm2* and accounted for its presence, we included a covariate (option –c) for binary information on the presence of *Pm2* based on haplotype-specific PCR screening.

Random subsets were produced using R package tibble 3.1.8 (Müller and Wickham 2022). Subsets of geographical origin were based on countries of origin.

The physical position of previously described *Pm* genes was either taken from the corresponding publication when available or estimated by blasting the flanking markers using BLASTN against Chinese Spring RefSeq v1.0 as a reference.

Accessions that possibly carry the causal genes of a resistance-associated region were determined by 1) having at least 50% of the resistance-associated SNPs within an associated region and 2) being resistant to the respective isolate (< = 20% leaf coverage).

## Results

**Fig. 1:**
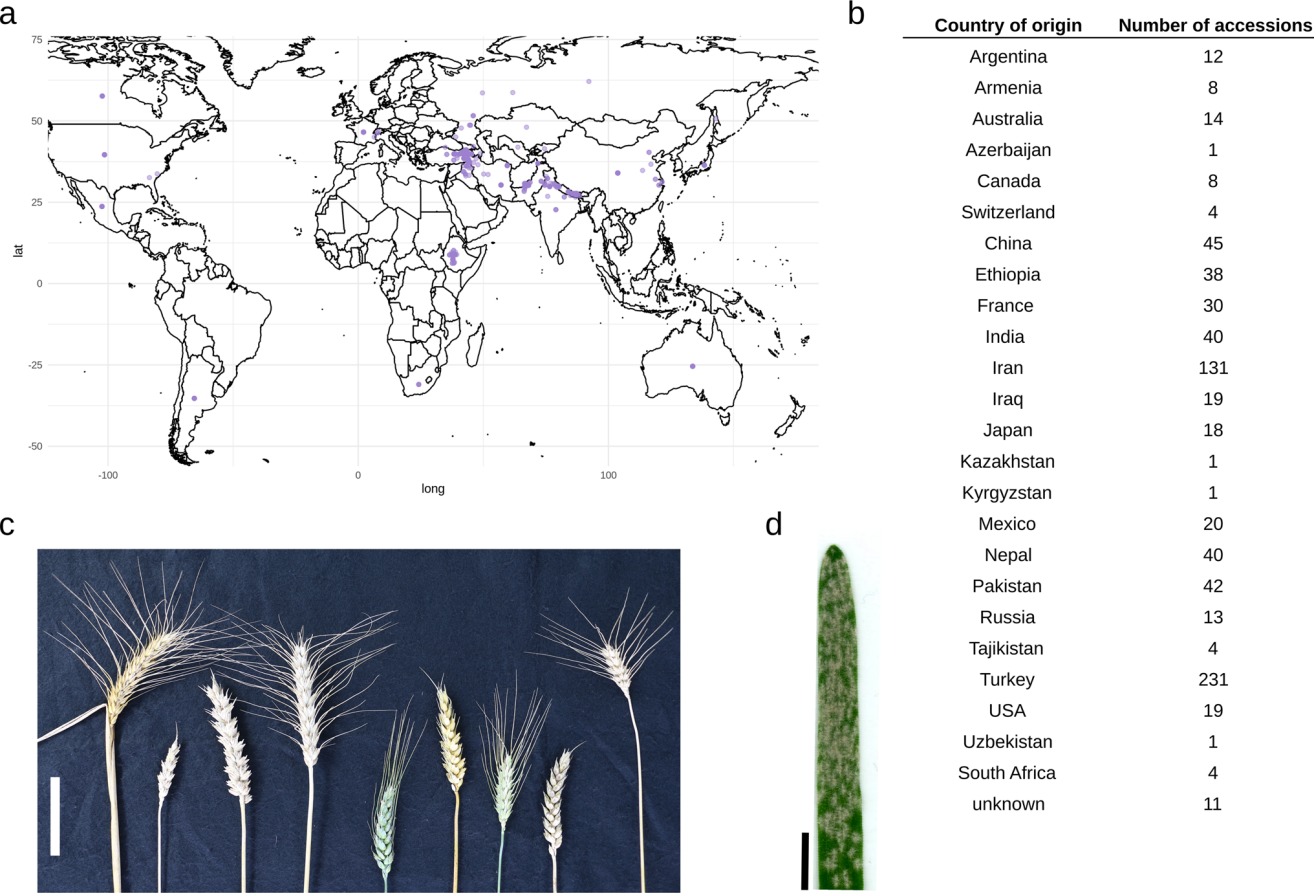
LandracePLUS panel diversity and powdery mildew symptoms: **a** World map with semitransparent purple dots representing the origin of each of the 744 of the 755 wheat accessions of the LandracePLUS panel with known origin, **b** number of accessions from the LandracePLUS panel per country of origin, **c** selection of diverse wheat spikes from the LandracePLUS panel. Scale bar, 5 cm, and **d** leaf of wheat cultivar “Kanzler” with powdery mildew symptoms. Scale bar, 1 cm (Color figure online)

We assembled a geographically diverse panel of 755 bread wheat accessions (Fig. 1a–c) based on the selective reduction of former collections used for allele mining of the powdery mildew resistance gene *Pm3* (Bhullar et al. 2009, 2010b). In the selection process, we focused on landraces and combined data on geographical origin with phenotypes of powdery mildew seedling stage resistance to six *Bgt* isolates. The resulting panel, hereafter LandracePLUS panel, contains 521 winter wheat and 234 spring wheat accessions, including 576 landraces, 162 older cultivars (acquisition date from 1946 to 2003, with the main part from 60s to 70s), seven research or breeding lines and 11 unknown accessions (Table S1). We used the LandracePLUS panel, which covers a broad geographical distribution, with a focus on accessions originating from the Middle East (Fig. 1a, b), to detect genetic loci associated with resistance to the powdery mildew pathogen (Fig. 1d).

### Diversity analysis of the LandracePLUS panel reveals unexplored genetic diversity

A total of 29,965 high-quality, polymorphic SNPs derived from genotyping with the TaBW35K SNP array were used for diversity analysis. All these SNPs had known chromosomal positions based on the Chinese Spring reference genome assembly RefSeq v1.0 (IWGSC 2018), including 18,610 regular SNPs and 11,355 OTVs, i.e., markers that detect both presence–absence polymorphisms and nucleotide polymorphisms. The 29,965 markers were distributed across the wheat genome, similar to earlier findings (Liu et al. 2017; Alemu et al. 2021; Govta et al. 2022), with lower coverage of genome D compared to the A and B genomes: 11,684 (39.0%) markers on the A-genome, 13,589 (45.3%) on the B-genome and 4692 (15.7%) on the D-genome. On average, 1,427 markers were assigned per chromosome, resulting in an average density of one marker each 483 Kbp. The least markers were assigned to chromosome 1D and most to chromosome 2B, with 497 and 2,465 markers, respectively.

**Fig. 2:**
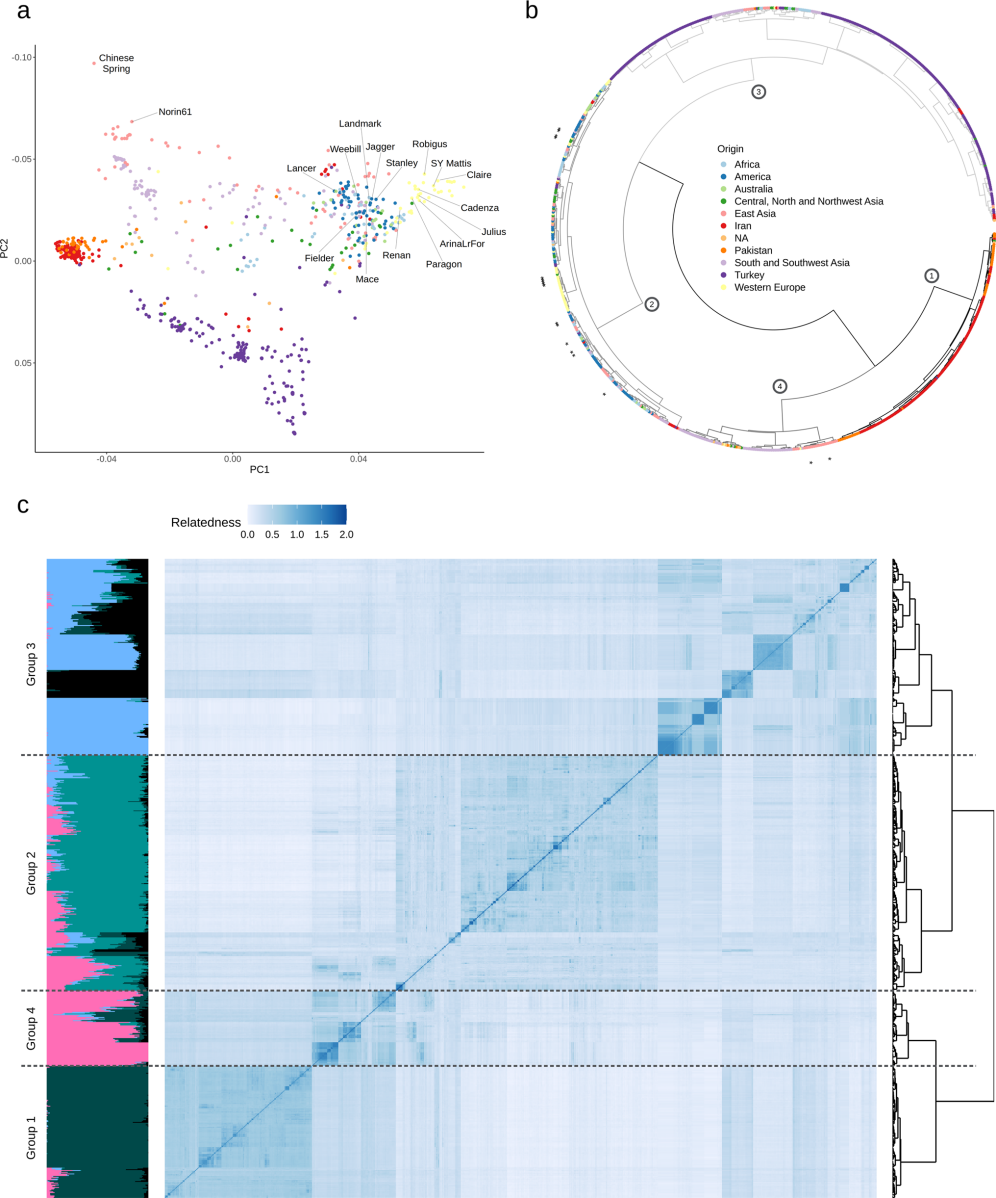
Genetic diversity and kinship analysis of the LandracePLUS panel: **a** PCA from 27,337 SNPs including high-quality sequenced wheat accessions labeled in the figure. PC1 = 8.4%, PC2 = 5.1%. Colors refer to the geographical origin of accessions and are indicated in **b**. **b** Dendrogram of a hierarchical clustering analysis from 27,337 SNPs including high-quality sequenced accessions indicated with stars. Colors represent the geographical origin of wheat accessions. Circled numbers on nodes refer to groups 1 to 4 when dividing into four clusters. **c** Alignment of Admixture plot, kinship matrix and dendrogram for the 755 wheat accessions based on 29,965 SNPs. The Admixture plot shows K = 5, where colors represent ancestral populations. In the kinship matrix, more saturated shades of blue indicate stronger relatedness. Dashed lines separate the four groups based on hierarchical clustering (Color figure online)

For a better interpretation of the observed genetic diversity within the LandracePLUS panel, wheat accessions with high-quality genome sequences, namely the 10 + wheat reference genomes (IWGSC 2018; Walkowiak et al. 2020), Fielder (Sato et al. 2021) and Renan (Aury et al. 2022) were included in the analysis with 27,337 out of the 29,965 SNPs that mapped unambiguously to chromosomes of these genomes. A PCA (Fig. 2a) and a dendrogram based on hierarchical clustering analysis (Fig. 2b) revealed four genetic clusters correlated with geographical origin overall. Group 1 was dominated by accessions from Iran and Pakistan, while group 2 was composed of accessions of mixed origin (Ethiopia, South Africa, USA, Canada, Mexico, Argentina, France, Switzerland, Kazakhstan, Kirgizstan, Tajikistan, Uzbekistan, Armenia and Russia). Group 3 predominantly contained accessions from Turkey, and group 4 was dominated by South, Southwest and East Asia (Iraq, Azerbaijan, India, Nepal, China and Japan). Most accessions with reference genomes available clustered in group 2 of the LandracePLUS panel, while Norin61 and Chinese Spring were on the edge of group 4 (Fig. 2a).

When highlighting the different types of accessions in the PCA, landraces covered almost the entire genetic diversity range of the LandracePLUS panel. In contrast, the 162 cultivars clustered mainly with group 2 and partially with group 4 (Fig. S2a). Therefore, landraces were genetically more diverse than cultivars and clustered apart from them. However, this was not always the case, as shown by the example of Ethiopian landraces, which clustered with cultivars in group 2 (Fig. S2b, Fig. 2a), suggesting that Ethiopian landraces have substantially contributed to breeding programs. The close clustering of accessions from all over the world described above (Fig. 2a, b) is likely driven by this separation between landraces and cultivars. As most of these geographically diverse accessions clustering together are cultivars, their genetic similarity is not derived from their origin but their breeding status as cultivars.

We assessed population structure performing hierarchical clustering on the 29,965 SNP set (excluding the high-quality genome sequences) and compared it to a kinship matrix and Admixture analysis (Fig. 2c). The found clusters reflected the four groups described earlier, except for a shift of several accessions of diverse origin from group 3 to group 2. Estimated ancestral populations K = 5 revealed a good fit for the LandracePLUS panel’s diversity into four groups (Fig. S1). Yet, the kinship matrix revealed additional subdivisions within Groups 3 and 4.

To assess whether the LandracePLUS panel represents a similar genetic diversity compared to former studies on wheat germplasm, we compared our data with a collection of 632 landraces that were genotyped with the TaBW280K SNP array (Rimbert et al. 2018), which contains all markers that were used for the LandracePLUS panel (Balfourier et al. 2019). A PCA with 29,965 filtered SNPs (Fig. S3) showed that the LandracePLUS panel covers the genetic diversity of this collection and further revealed unexplored genetic diversity absent in the study of Balfourier and colleagues (Balfourier et al. 2019). This additional diversity was mainly in groups 1 and 3, comprising Turkish, Pakistani and Iranian landraces.

Taken together, the LandracePLUS panel is a diverse selection of wheat accessions with a pronounced diversity of landraces compared to cultivars and high-quality sequenced genomes. Furthermore, the panel covers earlier found genetic diversity and additionally expands it, mainly with Turkish, Pakistani and Iranian landraces.

### Differential lines reveal diverse virulence in a set of ten powdery mildew isolates

**Fig. 3:**
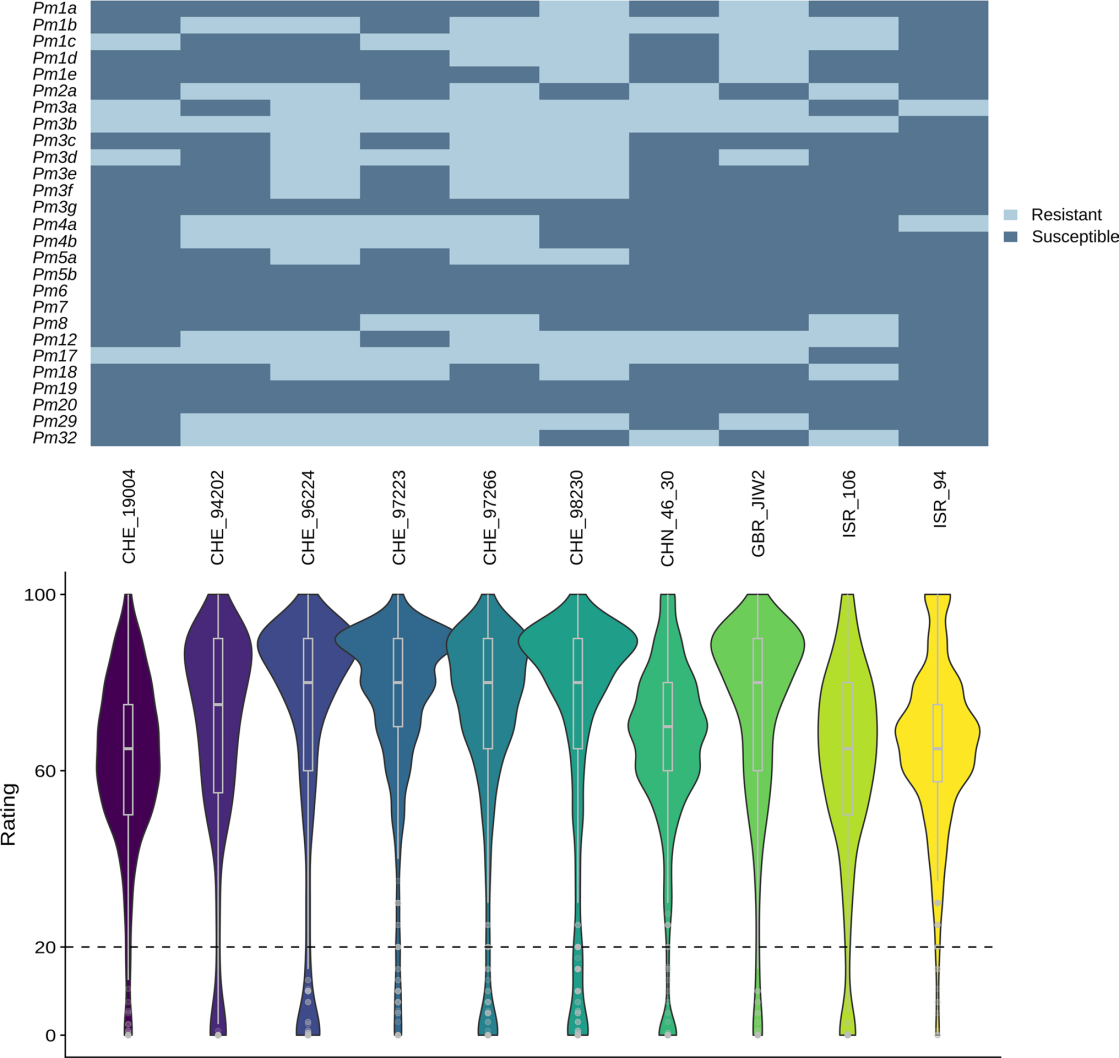
Virulence patterns of powdery mildew isolates and resistance distribution in the LandracePLUS panel. Virulence pattern of the ten powdery mildew isolates on differential lines with known *Pm* genes are shown as a heatmap on the top. On the bottom, the virulence pattern of the ten powdery mildew isolates on the LandracePLUS panel is depicted via violinplots and boxplots, highlighting median, 25 and 75 percentiles. The defined resistance threshold of 20 is indicated by a dashed line. Differential lines for the 27 *Pm* genes are listed in Table S2

To maximize the chances of finding novel resistance genes, we first tested ten random isolates on a global collection of 27 differential lines, including NILs and donors of cloned or genetically described *Pm* genes (Fig. 3). On average, isolates were avirulent on ten out of 27 differential lines. While isolates CHE_96224, CHE_97266 and CHE_98230 were avirulent on 16, 18 and 16 resistant differential lines, respectively, isolates CHE_19004 and ISR_94 were more virulent, with five and two resistant lines, respectively. Each isolate had a distinct virulence pattern, reflecting that the ten chosen mildew isolates represent broad diversity in virulence, confirmed by haplotype analysis of molecularly cloned *Avrs* (Table S4).

Taken together, the resistance patterns of the differential lines reveal a phenotypically diverse set of ten powdery mildew isolates.

### The LandracePLUS panel shows varying resistance to ten wheat powdery mildew isolates

The response of the 755 wheat accessions to the ten powdery mildew isolates revealed an overall susceptibility, with an average of 56 resistant accessions (rating <  = 20) per isolate (Fig. 3). Considering the response to individual isolates, only 35, 36, 40 and 17 wheat accessions were resistant to CHE_19004, CHE_97223, GBR_JIW2 and ISR_94, respectively. This reflected the broad virulence of CHE_19004 and ISR_94 already found in the differential lines, while CHE_97223 and GBR_JIW2 seemed to be more virulent on the LandracePLUS panel compared to the set of differential lines. On the other side, 91 and 89 wheat accessions were resistant to CHE_96224 and CHE_98230, respectively, which were the most avirulent isolates on the differential lines along with CHE_97266.

Phenotypic Pearson’s correlation between the isolates based on the set of differential lines corroborated the diversity of virulence (Fig. S4a), with an average correlation coefficient of 0.31. The most similar isolates were CHE_96224 and CHE_97266, as well as CHE_98230 and GBR_JIW2, with a correlation of 0.69, while the two most diverging, though not significantly, were ISR_106 and ISR_94 with a negative correlation of 0.18. Pearson’s correlation between the isolates based on the LandracePLUS panel showed patterns resembling the differential line phenotypes (Fig. S4b). While the overall relation between isolates was similar, several correlation coefficients differed notably, mostly involving ISR_106, GBR_JIW2 and CHE_98230. This deviation could be caused by different contents of *Pm* genes in the LandracePLUS panel compared to the differential set. With a coefficient of 0.36, however, the average correlation was similar. The diversity in resistance reactions observed within the LandracePLUS panel against these *Bgt* isolates suggests highly diverse effector content, highlighting the chances of finding novel resistance loci in the LandracePLUS panel.

No wheat accession showed resistance to all ten isolates, while nine wheat accessions were resistant to nine of the isolates. On average, excluding accessions susceptible to all tested isolates, wheat accessions were resistant to three isolates.

We predicted the presence of known *Pm* genes in accessions of the LandracePLUS panel that matched the pattern of the corresponding *Pm*-containing differential line. For example, 23 accessions of the LandracePLUS panel had the same resistance pattern as the *Pm2a* differential line and, hence, are good candidates for containing *Pm2a*. Indeed, 15 of these 23 accessions contained *Pm2* according to haplotype-specific markers (Manser et al. 2021) (Table S1). However, the potential presence of several *Pm* genes in the same accession can mask the resistance pattern of a specific *Pm* gene. This limits the use of differential lines to analyze overlapping resistance patterns and to predict specific genes. Nevertheless, the information on resistance patterns can be a useful tool to narrow down candidate accessions for the presence of a *Pm* gene of interest.

### The LandracePLUS panel shows isolate-dependent heritability of mildew response phenotypes and low correlation between phenotype and genetic relatedness

We calculated the phenotypic heritability using a linear mixed model approach. The effect of the wheat genotype on mildew resistance was isolate-dependent, between 0.38 for ISR_94 and 0.79 for CHE_96224, and with a batch effect of 0.48 and 0.12, respectively (Table S5). This suggests that the observed response to CHE_96224 is highly reproducible, while the batch seemed to have a strong influence on resistance reaction for ISR_94. Half of the isolates (CHE_94202, CHE_96224, CHE_97223, CHE_97266 and GBR_JIW2) had a heritability of 0.7 or higher, while ISR_94 was the only mildew isolate with a value below 0.5, possibly indicating a mixture of powdery mildew races. Accordingly, we removed isolate ISR_94 from all subsequent analyses. To account for this batch effect for the remaining isolates, and since the described nature of *R* genes can be considered a binary one—resistant or susceptible—we decided to transform the phenotypic scoring values of 0 to 100 to these two categories, with a threshold of 20 or higher for susceptibility. Thus, differences between batches are weighted less, providing more reliable phenotypes and GWAS results.

Using these categorized phenotypes, we further tested the correlation between the genetic relatedness and the phenotype using a Mantel test (Mantel and Valand 1970). There were only slight differences between the ten different isolates, and the correlations were close to zero, with Mantel r values ranging from − 0.001 to 0.029, although not significant for most isolates (Table S6). This suggests that the applied approach of assembling the LandracePLUS panel minimized the effect of population structure on trait variation. The LandracePLUS panel should, therefore, provide improved power when conducting GWAS (Myles et al. 2009).

### Association studies for seedling resistance to wheat powdery mildew in the LandracePLUS panel reveal previously cloned Pm genes as well as possibly novel genes

**Fig. 4:**
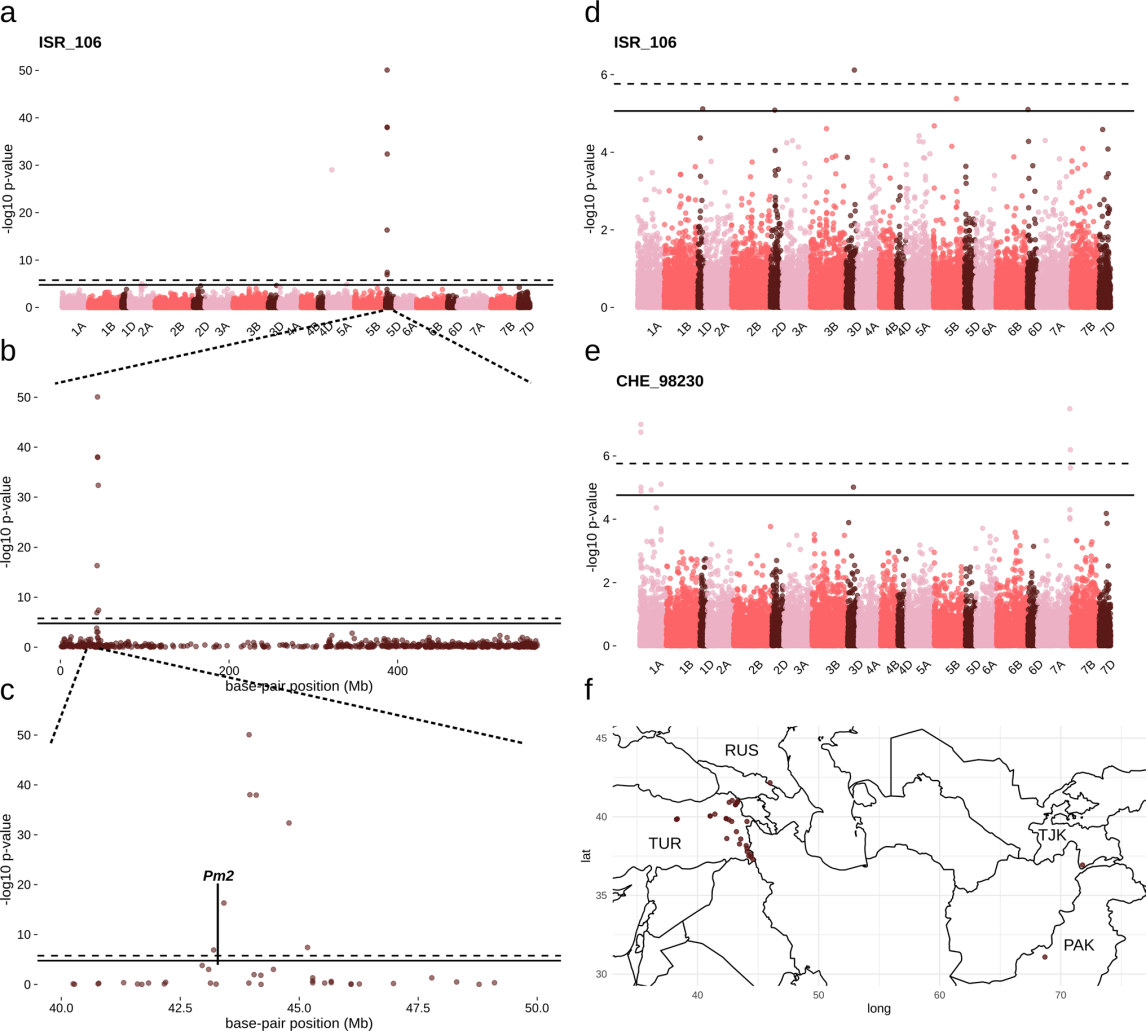
Analysis of the region associated with powdery mildew resistance on chromosome 5D. Manhattan plot for GWAS with all wheat accessions infected with isolate ISR_106 showing **a** all 21 chromosomes, **b** chromosome 5D and **c** the region of 40 to 50 Mb on chromosome 5D. The locus of the Chinese Spring version of *Pm2* with a partially deleted gene (Sánchez-Martín et al. 2016) is indicated with a line. **d** Manhattan plot for GWAS with ISR_106 when adding a covariate for *Pm2* presence in wheat accessions. **e** Manhattan plot for the *Pm2* virulent isolate CHE_98230. Solid lines represent the threshold for false discovery rate, and dashed lines for Bonferroni correction. **f** Map with semitransparent brown dots depicting the origin of landraces that contain *Pm2*. Countries of origin are abbreviated with the three-letter country code of ISO 3166

We conducted GWAS with the phenotyping data obtained for each of the nine powdery mildew isolates on the LandracePLUS panel with a MAF of 1%. We first tested for a good fit of the univariate linear mixed model based on QQ plots, which was confirmed for all isolates except CHE_97223 (Fig. S5). GWAS of the other eight isolates revealed five genomic regions associated with wheat mildew resistance on chromosomes 1A, 2B, 5D, 7A and 7D (Table 1, Fig. S6). To account for LD, we defined a peak region by adding the average LD decay distance per subgenome from a recent study in wheat (Liu et al. 2023) to either side of the peak SNP. The most significant peak was located on chromosome 5D for the *AvrPm2*-containing *Bgt* isolates CHE_94202, CHE_96224, CHE_97266, CHN_46_30 and ISR_106. When focusing on one representative *AvrPm2*-containing isolate, ISR_106, the mildew resistance association spanned the region from 40,919,172 to 46,974,772 bp of the short arm of chromosome 5D of Chinese Spring (Fig. 4a–c) and included the *Pm2* resistance gene locus (Sánchez-Martín et al. 2016). To test if the presence of *Pm2* was responsible for the significant association, we screened the LandracePLUS panel with a *Pm2* haplotype-specific marker (Manser et al. 2021) and found that out of 66 wheat accessions resistant to ISR_106, 31 contained *Pm2*. In the whole panel, 39 accessions contained *Pm2*, of which 34 were landraces (Table S1). Most of these 34 accessions were from Turkey, with three landraces from Russia, Pakistan and Tajikistan (Fig. 4f). GWAS with a covariate for *Pm2* presence resulted in the loss of the significant peak (Fig. 4d), corroborating that the peak was indeed caused by *Pm2*. Sequencing of the amplified *Pm2* locus in four randomly selected landraces that were positive for the haplotype marker uniformly revealed the presence of the known allele *Pm2a* (Sánchez-Martín et al. 2016).

**Table 1:** Powdery mildew-associated regions detected in the LandracePLUS panel

Chromosome	Position	Isolate	beta	*Pm* genes described on specific chromosome arm/chromosome
1AS	306,708–12,377,308	CHE_98230	−0.14	***Pm3***(1)*, Pm17*(2)*, Pm25*(3)
1BL	610,940,301–615,710,501	CHE_96224*	−0.08	*Pm28*(4), *Pm39*(5)
2BL	707,987,645–722,628,991**	CHE_96224	−0.20	*Pm6*(6)*, Pm33*(7)*, ****Pm51***(8)*, Pm52*(9)*, Pm57*(10)*, Pm62 (11), ****Pm63***(12)*, Pm64*(13)*, ****PmKN0816***(14)*, ****PmLS5082***(15)*, ****PmQ***(16)*, PmY39*(17)*, ****pmYN99102***(18)*, MlAB10*(19)*, Mlzec1*(20), ***PMCG15-009***(21)
3BL	667,372,099–672,142,299	CHN_46_30*	−0.05	*Pm41*(22)*, PmHNK*(23)
3BL	701,911,458–706,681,658	CHE_97266*	−0.15	*Pm41*(22)*, PmHNK*(23)
5BL	647,102,735–651,872,935	CHN_46_30*	−0.21	*Pm36*(24)*, Pm53*(25)*, PmAS846*(26)*, Ml3D232*(27)
5DS	40,919,172–46,974,772	CHE_94202 CHE_96224 CHE_97266 CHN_46_30 ISR_106	−0.37 −0.26 −0.23 −0.39 −0.39	***Pm2***(28)*, ****Pm46*** (29)*, Pm55*(30)
7AL	717,179,300–733,267,996	CHE_94202 CHE_97266 CHE_98230 GBR_JIW2	−0.22 −0.22 −0.24 −0.35	*Pm1* (31)*, Pm9* (32)*, Pm37*(33)*, ****Pm59***(34)*, ****Pm60***(35)*, ****PmG16***(36)*, ****MlIw72***(37)*, ****Mlm2033***(38)*, ****Mlm80***(38)*, ****mlRd30***(39)*, ****MlUM15***(40)*, PmU*(41)*, PmTb7A.1*(42)*, PmTb7A.2*(42)*, ****MlIW172***(43)*, ****MlWE18***(44)
7BS	68,921,370–73,691,570	CHE_96224*	−0.12	*Pm40(45), Pm47(46), mljy(47), mlsy(47)*
7DL	631,068,212–637,123,812	GBR_JIW2	−0.10	*Pm19(48), Pm29(49)*

Physical positions of GWAS peaks on Chinese Spring and the isolates detecting them. Values for beta are shown for the SNP of each peak with the strongest effect size. If several isolates produce the same peak, beta is listed for each. *Pm* genes are listed that were described on the same chromosomal arm or the same chromosome without a specified arm. *Pm* genes described in an overlapping genomic interval with the GWAS peak are highlighted in bold. * Usage of a covariate for *Pm2* in the regarding association. ** Region of significant association without extension based on LD decay. (1) Yahiaoui et al. 2004, (2) Singh et al. 2018, (3) Shi et al. 1998, (4) Peusha et al. 2004, (5) Lillemo et al. 2008, (6) Wan et al. 2020, (7) Zhu et al. 2005, (8) Zhan et al. 2014, (9) Wu et al. 2019, (10) Dong et al. 2020, (11) Zhang et al. 2018, (12) Tan et al. 2019, (13) Zhang et al. 2019, (14) Wang et al. 2021, (15) Wu et al. 2022, (16) Li et al. 2020a, b, (17) Zhu et al. 2006, (18) Mu et al. 2022, (19) Maxwell et al. 2010, (20) Mohler et al. 2005, (21) Zhang et al. 2023, (22) Li et al. 2020a, (23) Xu et al. 2010, (24) Blanco et al. 2008, (25) Petersen et al. 2015, (26) Xue et al. 2012, (27) Zhang et al. 2010, (28) Sánchez-Martín et al. 2016, (29) Gao et al. 2012, (30) Zhang et al. 2016, (31) Hewitt et al. 2021, (32) McIntosh et al. 2013, (33) Perugini et al. 2008, (34) Tan et al. 2018, (35) Zou et al. 2018, (36) Ben-David et al. 2010, (37) Ji et al. 2008, (38) Yao et al. 2007, (39) Singrün et al. 2004, (40) Worthington et al. 2014, (41) Qiu et al. 2005, (42) Chhuneja et al. 2015, (43) Ouyang et al. 2014, (44) Han et al. 2009, (45) Luo et al. 2009, (46) Xiao et al. 2013, (47) Huang et al. 2002, (48) Lutz et al. 1995, (49) Zeller et al. 2002

In addition to *Pm2*, we detected a peak on chromosome 1AS for CHE_98230, which spanned the genomic region from 306,708 to 12,377,308 bp (Table 1, Fig. S6) and contains the locus of the cloned gene *Pm3*. Earlier studies have shown that out of 89 resistant accessions in the LandracePLUS panel, 21 contained functional *Pm3* alleles, with 12 and five accessions containing *Pm3c* and *Pm3b*, respectively (Table S1) (Bhullar et al. 2009, 2010a, b). Therefore, we propose that the peak is caused by these functional *Pm3* alleles.

A third, very large region on chromosome 2BL significantly showed CHE_96224 resistance-associated SNPs from 707,987,645 to 722,628,991 bp (Table 1, Fig. S6). Based on our criteria, this region was defined as three independent peaks. Upon closer inspection, the peaks are, however, only separated by small intervals showing no association. We therefore decided to consider them a single mildew-associated region. Indeed, it was shown that an introgression on chromosome 2BL is present in the wheat gene pool, most likely derived from the diploid wild relative *Triticum timopheevii* (Walkowiak et al. 2020; Keilwagen et al. 2022). This introgression is potentially present in the LandracePLUS panel. Many candidate *Pm* genes have already been described in this genomic interval, including *Pm51* (Zhan et al. 2014), *Pm63* (Tan et al. 2019), *PmKN0816* (Wang et al. 2021), *PmLS5082* (Wu et al. 2022), *PmQ* (Li et al. 2020b), *pmYN99102* (Mu et al. 2022) and *PmCG15-009* (Zhang et al. 2023).

We further detected a peak on chromosome 7AL for CHE_94202, CHE_97266, CHE_98230 and GBR_JIW2 that spanned from 717,179,300 to 733,267,996 bp (Table 1, Fig. S6). Possible candidate genes in this genomic interval include *Pm59* (Tan et al. 2018), *MlIw72* (Ji et al. 2008), *Mlm2033* (Yao et al. 2007), *Mlm80* (Yao et al. 2007), *MlUM15* (Worthington et al. 2014), *MlIw172s* (Ouyang et al. 2014), *PmG16* (Ben-David et al. 2010) and *mlRd30* (Singrün et al. 2004). Alleles of *Pm1* have also been mapped to chromosome 7AL (McIntosh et al. 2013). However, we could not confirm an overlap with our resistance-associated region because the *Pm1* locus is absent in Chinese Spring (IWGSC 2018), and based on blast search using the genomic sequence of *Pm1a* as query, this locus is also absent in all other chromosome-scale-assembled wheat varieties (Walkowiak et al. 2020; Sato et al. 2021; Aury et al. 2022; Athiyannan et al. 2022a).

**Fig. 5:**
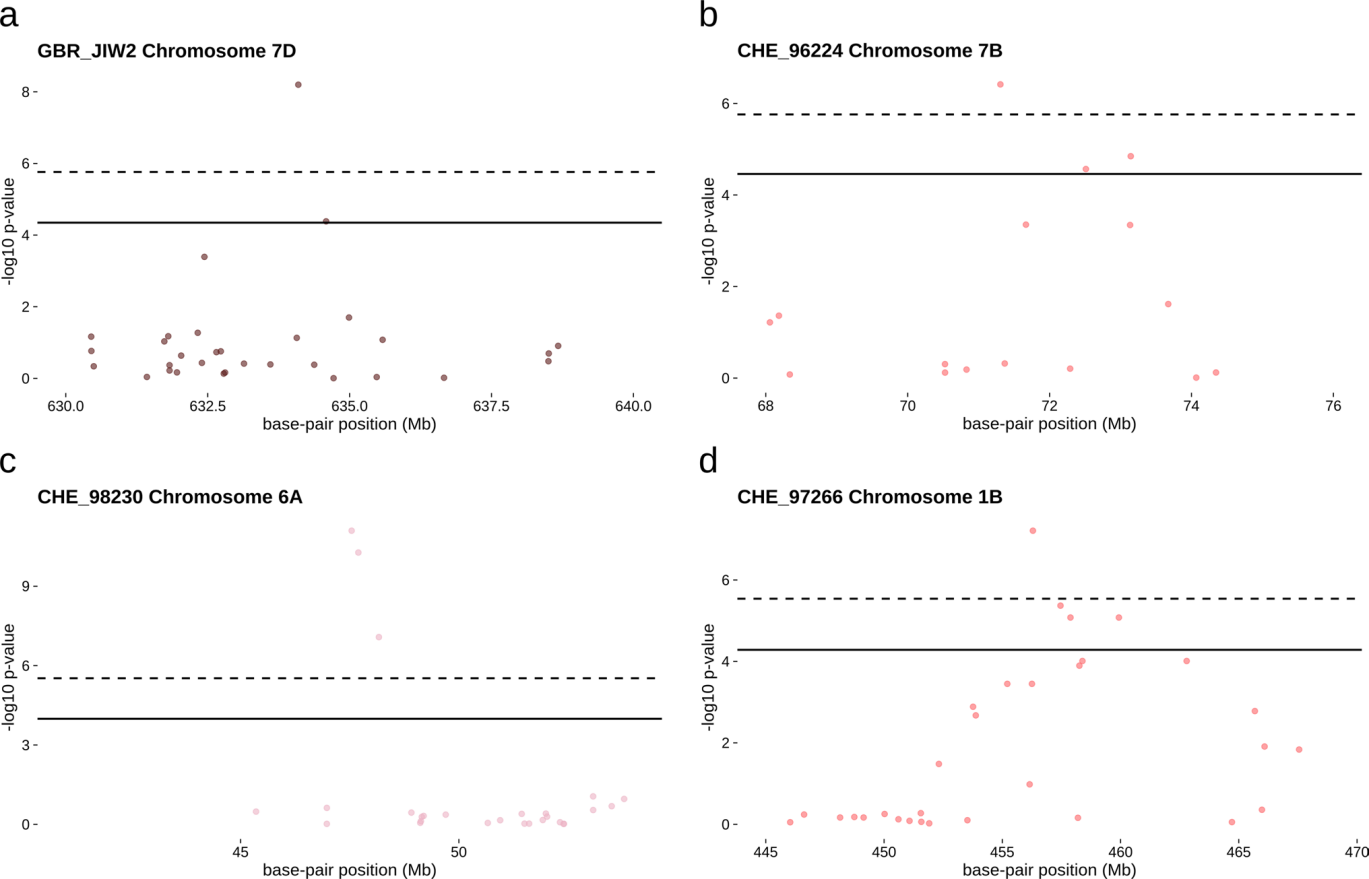
Manhattan plots of novel resistance-associated regions: **a** all phenotyped LandracePLUS panel accessions, **b** all phenotyped LandracePLUS panel accessions using a *Pm2* covariate, **c** subset of accessions from Pakistan and Iran, and **d** subset of accessions from Turkey with CHE_97266 representing the peak occurring for multiple isolates. Isolates and chromosomes are depicted in the top-left corner. Solid lines represent the threshold for false discovery rate and dashed lines for Bonferroni correction

Finally, we detected a peak on chromosome 7DL for GBR_JIW2 that spanned from 631,068,212 to 637,123,812 bp (Table 1, Fig. S6, Fig. 5a), where no *Pm* gene has been described previously.

### Association studies with a Pm2 covariate reveal five additional and novel resistance-associated loci

We used the information on the presence of *Pm2* in LandracePLUS panel accessions to detect further resistance associations masked by the *Pm2* gene. Incorporating the covariate in GWAS for isolates with the *Pm2* peak, we detected five additional associations on chromosomes 1B, 3B, 5B and 7B (Table 1, Fig. S7, Fig. 5b). Two of these peaks appeared for CHE_96224, on chromosome 1BL, spanning from 610,940,301 to 615,710,501 bp and on chromosome 7BS from 68,921,370 to 73,691,570 bp. CHE_97266 showed an association on chromosome 3BL from 701,911,458 to 706,681,658 bp, and the last two associations belonged to CHN_46_30, with a peak on chromosome 3BL from 667,372,099 to 672,142,299 bp and another on chromosome 5BL from 647,102,735 to 651,872,935 bp. These associations occur in regions where no *Pm* gene has been reported earlier, highlighting the utility of using a known *Pm* gene as a covariate to discover new resistance loci in GWAS.

### Pm4 alleles are widely present in the LandracePLUS panel but are not revealed by association studies

Based on the resistance of differential lines carrying genes *Pm4a* or *Pm4b* to five and four of the nine isolates, respectively (Sánchez-Martín et al. 2021), we expected to detect the cloned gene *Pm4* in the LandracePLUS panel. Haplotype-specific markers detected the presence of the *Pm4* haplotype in 62 accessions. However, no peak was produced at the *Pm4* locus on chromosome 2AL for any isolate. To explain this missing association despite the broad presence of *Pm4*, we sequenced the locus in all 62 accessions, of which 51 and four accessions contained the non-functional alleles *Pm4f* and *Pm4g*, respectively (Sánchez-Martín et al. 2021), and four accessions with an undescribed allele, hereafter *Pm4_42460*, while the functional alleles *Pm4b* and *Pm4d* only occurred once and twice, respectively. The undescribed allele resembled *Pm4f*, except for one amino acid change in position 421 of splicing variant V1 (L421P). While allele *Pm4g* was present in accessions of diverse geographical origin, 42 out of 51 accessions carrying *Pm4f* and all four accessions containing *Pm4_42460* were Turkish landraces. Our findings suggest that the non-functional alleles *Pm4_42460* and *Pm4f* originated in Turkey.

Taken together, we find the presence of known *Pm* genes with GWAS of the LandracePLUS panel, giving insights into their geographic distribution and potential origin. In addition, we discovered six genomic regions where no *Pm* gene had been described earlier.

### Utilizing subsets of the LandracePLUS panel discovers novel loci that are associated with powdery mildew resistance of distinct geographical origin

Some resistance genes are likely present only in certain groups of the LandracePLUS panel, e.g., accessions with similar geographical origins like for the *Pm2* resistance gene. Accordingly, we first tested if subsets of the panel based on such groups were suitable to detect additional genomic regions associated with powdery mildew resistance. To assess the sensitivity of this approach, we used a re-sampling approach by creating 100 random subsets of 300, 200, 150 and 90 accessions from the LandracePLUS panel as input for GWAS with the *Pm2* avirulent isolate ISR_106. Since the MAF threshold of 1% used above would not filter out SNPs that occur only twice for a subset of 200 to 299 accessions, we adjusted the MAF threshold to 5% for all subsets below a sample size of 300. We found the *Pm2*-associated peak in 100%, 87%, 78% and 52% of cases for the four subset sizes, respectively. Therefore, we considered subsets of a minimum of 150 to 200 accessions suitable for detecting loci associated with a major resistance gene in the LandracePLUS panel.

In our subsetting approach, we created sets of accessions based on their geographical origins. A subset of landraces from Pakistan and Iran showed a good fit to the GWAS model only for the mildew isolate CHE_98230 (Fig. S8), revealing five additional peaks compared to the full LandracePLUS panel, located on chromosomes 2B, 5A, 5B, 5D and 6A (Table 2, Fig. S9, Fig. 5c). While the peak on chromosome 2BL was located in a genomic region that has been described to contain the resistance gene *Pm51* (Zhan et al. 2014) and the peak on chromosome 5BL has been implicated earlier with the resistance genes *Pm53* (Petersen et al. 2015) and *Ml3D232* (Zhang et al. 2010), the associated regions on chromosomes 5AL from 679,959,567 to 692,030,167 bp and on 5DS from 26,487,899 to 32,543,499 bp have not been associated with powdery mildew resistance before to our knowledge (Table 2). The last resistance-associated region, on chromosome 6AS, spanned the region from 41,508,045 to 53,578,645 (Fig. 5c). On chromosome arm 6AS, only *Pm21* and *Pm56* have been described. *Pm21* originated and was cloned from the diploid grass *Dasypyrum villosum* (He et al. 2018; Xing et al. 2018). The gene was introduced in the hexaploid gene pool in China through a translocation line T6AL.6VS in the late 1980s (Chen et al. 1995). However, the translocated arm from *D. villosum* does not recombine with wheat homeologs (He et al. 2017). Hence, we expect to see a broad association covering the short arm of chromosome 6A in the case of *Pm21* detection. Further, *Pm21* was described to confer broad-spectrum resistance (He et al. 2017), whereas we observed the association for only one out of ten isolates. Therefore, we assume that the causal gene in our resistance-associated region differs from *Pm21*. Similarly, *Pm56* was only recently introduced as a translocation line 6AL.6RS from rye (Hao et al. 2018) several years after we obtained and utilized the seeds of the initial collection. We conclude that this is a resistance-associated region on chromosome 6AS not previously described.

**Table 2:** Powdery mildew-associated regions detected in subsets of the LandracePLUS panel

Chr	Position	Subset	Isolate	beta	*Pm* genes described on specific chromosome arm/chromosome
1AL	539,831,968–551,902,568	Turkey (221)	CHE_98230	−0.14	*Pm25*(1)
1BL	453,908,676–462,314,398	Turkey (223, 222, 222, 217, 212)	CHE_94202 CHE_96224 CHE_97266 CHN_46_30 ISR_106	−0.22 −0.24 −0.26 −0.28 −0.26	*Pm28*(2), *Pm39*(3)
1BL	664,434,720–669,204,920	Turkey (222)	CHE_96224	−0.22	*Pm28*(2), ***Pm39***(3)
1DL	333,346,020–339,401,620	Turkey (217, 212)	CHN_46_30 ISR_106	−0.48 −0.48	*Pm10*(4)
2AL	534,734,122–546,804,722	Turkey (221)	CHE_98230	0.38	*Pm4*(5)*, Pm50*(6)*, Pm65*(7)*, pmX*(8)*, PmHo*(9)*, Ml92145E8-9*(10)*, PmHNK54*(11)*, PmLK906*(12)*, PmYm66*(13)*, PmSN15218*(14)
2AL	762,574,656–774,645,256	Turkey (221)	CHE_98230	0.84	*Pm4*(5)*, Pm50*(6)*, ****Pm65***(7)*, ****pmX***(8)*, PmHo*(9)*, Ml92145E8-9*(10)*, PmHNK54*(11)*, PmLK906*(12)*, PmYm66*(13)*, ****PmSN15218***(14)
2BL	737,551,805–742,322,005	Pakistan/Iran (167)	CHE_98230	−0.33	*Pm6*(15)*, Pm33*(16)*, ****Pm51***(17)*, Pm52*(18)*, Pm57*(19)*, Pm62 (20), Pm63*(21)*, Pm64*(22)*, PmKN0816*(23)*, PmLS5082*(24)*, PmQ*(25)*, PmY39*(26)*, pmYN99102*(27)*, MlAB10*(28)*, Mlzec1*(29), *PMCG15-009*(30)
3AL	643,922,092–655,992,692	Turkey (220)	GBR_JIW2	−0.16	*–*
4BL	610,988,411–615,758,611	Turkey (224)	CHE_97223	−0.12	*Pm7*(31), *Mld*(32), *PmTx45*(33)
5AL	472,287,696–484,857,509	Turkey (217)	CHN_46_30	0.16	*pm2026*(34)
5AL	679,959,567–692,030,167	Pakistan/Iran (167)	CHE_98230	0.28	*pm2026*(34)
5BL	375,685,467–380,455,667	Turkey (223)	CHE_94202	−0.20	*Pm36*(35)*, Pm53*(36)*, PmAS846*(37)*, Ml3D232*(38)
5BL	517,686,966–522,457,166	Pakistan/Iran (167)	CHE_98230	0.19	*Pm36*(35)*, ****Pm53***(36)*, PmAS846*(37)*, ****Ml3D232***(38)
5BL	656,831,925–661,602,125	Turkey (217)	CHN_46_30	0.19	*Pm36*(35)*, Pm53*(36)*, PmAS846*(37)*, Ml3D232*(38)
5DS	26,487,899–32,543,499	Pakistan/Iran (167)	CHE_98230	0.27	*Pm2*(39)*, Pm46* (40)*, Pm55*(41)
6AS	0–7,996,136	Turkey (224)	CHE_97223	−0.12	*Pm21*(42, 43)*, Pm56*(44)
6AS	41,508,045–53,578,645	Pakistan/Iran (167)	CHE_98230	−0.25	*Pm21*(42, 43)*, Pm56*(44)
7AL	611,542,221–623,612,821	Turkey (224)	CHE_97223	−0.09	*Pm1*(45)*, Pm9* (46)*, Pm37*(47)*, Pm59(48), Pm60*(49)*, PmG16*(50)*, MlIw72*(51)*, Mlm2033*(52)*, Mlm80*(52)*, mlRd30*(53)*, MlUM15*(54)*, PmU*(55)*, PmTb7A.1*(56)*, PmTb7A.2*(56)*, MlIW172*(57)*, MlWE18*(58)
7BL	701,729,427–706,653,524	Turkey (221)	CHE_98230	−0.16	***Pm5***(59)*, ****pmDHT***(60)*, ****PmSGD***(61)*, ****PmTm4***(62)*, ****pmHYM***(63)*, ****pmYBL***(64)*, ****mlxbd***(65)*, mljy*(66)*, mlsy*(66)
7DL	497,931,007–503,986,607	Turkey (223, 222, 217, 212)	CHE_94202 CHE_96224 CHN_46_30 ISR_106	−0.16 −0.15 −0.16 −0.19	*Pm19*(67), *Pm29*(68)

Physical positions of GWAS peaks on Chinese Spring, subset description with number of accessions in brackets and the isolates detecting them. Chr = chromosome. Values for beta are shown for the SNP of each peak with the strongest effect size. If several isolates produce the same peak, beta is listed for each. *Pm* genes are listed that were described on the same chromosomal arm or the same chromosome without a specified arm. *Pm* genes described in an overlapping genomic interval with the GWAS peak are highlighted in bold. (1) Shi et al. 1998, (2) Peusha et al. 2004, (3) Lillemo et al. 2008, (4) Tosa et al. 1987, (5) Sánchez-Martín et al. 2021, (6) Mohler et al. 2013, (7) Li et al. 2019, (8) Fu et al. 2013, (9) Komáromi et al. 2016, (10) Yu et al. 2018, (11) Xu et al. 2011, (12) Niu et al. 2008, (13) Hu et al. 2008, (14) Sun et al. 2022, (15) Wan et al. 2020, (16) Zhu et al. 2005, (17) Zhan et al. 2014, (18) Wu et al. 2019, (19) Dong et al. 2020, (20) Zhang et al. 2018, (21) Tan et al. 2019, (22) Zhang et al. 2019, (23) Wang et al. 2021, (24) Wu et al. 2022, (25) Li et al. 2020a, b, (26) Zhu et al. 2006, (27) Mu et al. 2022, (28) Maxwell et al. 2010, (29) Mohler et al. 2005, (30) Zhang et al. 2023, (31) Driscoll and Bielig 1968, (32) McIntosh et al. 2013, (33) Chao et al. 2019, (34) Xu et al. 2008, (35) Blanco et al. 2008,(36) Petersen et al. 2015, (37) Xue et al. 2012, (38) Zhang et al. 2010, (39) Sánchez-Martín et al. 2016, (40) Gao et al. 2012, (41) Zhang et al. 2016, (42) He et al. 2018, (43) Xing et al. 2018, (44) Hao et al. 2018, (45) Hewitt et al. 2021, (46) McIntosh et al. 2013, (47) Perugini et al. 2008, (48) Tan et al. 2018, (49) Zou et al. 2018, (50) Ben-David et al. 2010, (51) Ji et al. 2008, (52) Yao et al. 2007, (53) Singrün et al. 2004, (54) Worthington et al. 2014, (55) Qiu et al. 2005, (56) Chhuneja et al. 2015, (57) Ouyang et al. 2014, (58) Han et al. 2009, (59) Hsam et al. 2001, (60) Qie et al. 2019, (61) Xu et al. 2018, (62) Xie et al. 2017, (63) Wang et al. 2022, (64) Xu et al. 2020, (65) Jin et al. 2020, (66) Huang et al. 2002, (67) Lutz et al. 1995, (68) Zeller et al. 2002

Finally, we used the same geographical approach to investigate only Turkish landraces, revealing a good fit to the model for all isolates except CHE_19004 (Fig. S10). This subsetting resulted in the discovery of 15 additional peaks on chromosomes 1A, 1B, 1D, 2A, 3A, 4B, 5A, 5B, 6A, 7A, 7B and 7D (Table 2, Fig. S11). In earlier studies, two of the 15 regions have been described with powdery mildew seedling resistance. The associated region on chromosome 2AL from 762,574,656 to 774,645,256 bp covers three known *Pm* genes, while the peak on chromosome 7BL from 701,729,427 to 706,653,524 bp overlaps with eight previously described *Pm* genes (Table 2). The association on chromosome 1BL from 664,434,720 to 669,204,920 bp includes the *Pm39* locus. However, *Pm39*, also known as *Lr46* (Lillemo et al. 2008), is an adult plant resistance gene and not active at seedling stage, and we conclude that the detected association is caused by an unknown, novel gene. The remaining 12 loci do not overlap with previously described *Pm* genes and therefore depict good candidates for novel powdery mildew resistance loci (Table 2).

Taken together, subsets based on geographical origin revealed 16 genomic regions where no *Pm* genes have been described previously, suggesting that these genes arose in the respective countries Pakistan, India and Turkey.

### Candidate genes of five novel resistance loci include putative NLRs, serine/threonine kinases, a C2H2-type zinc finger and F-box-like proteins with leucine-rich repeat (LRR) domains

We investigated five of the 22 novel resistance loci more closely to get an insight into possible candidate genes (Fig. 5, Table S7). We chose the most significant peak with at least two significantly associated SNPs from GWAS of: 1) the full LandracePLUS panel, 2) the panel using the *Pm2* covariate and 3) the Pakistan/Iran subset, on chromosomes 7DL, 7BS and 6AS, respectively. From the subset of Turkish landraces, we chose the highly significant single SNP association on chromosome 1DL and the peak on chromosome 1BL that occurred for five isolates, suggesting a more broad-spectrum resistance. For the associated region on chromosome 7DL, candidates annotated on Chinese Spring were ten putative NLRs, while the associated region on chromosome 7BS contained an annotation for a C2H2-type zinc finger and one F-box-like protein with an LRR domain. All associated regions except for 1BL had annotations for one serine/threonine kinase, the only candidate for the peak on chromosome 1DL. Both peaks on chromosome 6AS and 1BL contained annotations for one putative NLR, and the peak on chromosome 6AS included four additional F-box-like proteins with LRR domains (Fig. 5, Table S7). Further validation studies are necessary to confirm the resistant nature of these candidate genes. Due to the possibility of missing genes in the associated regions in the reference genome Chinese Spring, we also provide a list of candidate genes in all chromosome-scale-assembled wheat varieties (Supplementary_file4) (White et al. 2024). While Chinese Spring has 298 predicted genes in the five associated regions, the other 12 varieties ranged between 324, for Renan, and 471, for Lancer.

### Novel resistance loci for breeding programs

We analyzed the gene pool of the 20 most modern cultivars of the LandracePLUS panel (registered between 1990 and 2003) to evaluate whether the 22 potentially novel powdery mildew genes are present in elite material or potentially novel in this gene pool (Supplementary_file3). For genomic regions on chromosomes 5BL (derived from the LandracePLUS panel using a *Pm2* covariate) and 6AS (from the Pakistan/Iran subset), the most modern cultivars contained less than 50% of the resistance-associated SNPs, suggesting that the underlying resistance genes are likely not present in these cultivars. For six of the 22 associations, including the peaks on chromosomes 7DL and 1BL (described above in detail), some of the cultivars contained at least 50% of the resistance-associated SNPs, but none showed resistance toward the respective mildew isolate. We conclude that the underlying resistance genes are not present in this germplasm, at least not as active, resistance-conferring alleles. These findings suggest that the resistance loci have not been transferred to the modern gene pool from landraces. However, investigation of more recent cultivars would be needed to confirm these results.

Resistance-associated SNPs of the remaining 14 associated regions, including the peaks on chromosomes 7BS and 1DL discussed extensively, were present in the most modern germplasm. However, few accessions that harbored the alleles were resistant. Thus, the underlying resistance-conferring alleles seem to be partially present, meaning that breeders could integrate the corresponding cultivars we highlighted (Supplementary_file3) directly in their programs, avoiding possible yield penalties due to linkage drag. However, the combination of resistance and presence of resistance-associated SNPs occurred only for the three cultivars TRI 17181, TRI 17284 and TRI 16947. While these cultivars are attractive resistance-breeding candidates, it is difficult to dissect which detected regions are causing the observed resistance and, therefore, actually contain causative alleles.

## Discussion

### The LandracePLUS panel harbors untapped genetic diversity originating mainly from Turkish, Pakistani and Iranian landraces

We assembled a diverse panel of 755 bread wheat accessions with a focus on landraces. A FIGS approach laid the foundation for a trait-customized collection of accessions with potentially high selection pressure for powdery mildew resistance. A second step of selective reduction based on geographical origin resulted in the LandracePLUS panel. Mantel tests revealed a small impact of the population structure on powdery mildew resistance variation, reflecting the successful outcome of our targeted panel assembly. The resulting improved power of GWAS (Myles et al. 2009) facilitated the detection of novel *Pm* genes in the LandracePLUS panel.

The LandracePLUS panel revealed untapped genetic diversity in Turkish, Iranian and Pakistani landraces. It showed four main genetic clusters that correlate with the geographical origin, similar to a study which showed that wheat accessions from the Caucasus region, as well as Central, South and East Asian, are more diverse compared to other regions in the world (Balfourier et al. 2019). Landraces covered almost the full diversity of the LandracePLUS panel, whereas cultivars were limited to one cluster. This cluster also included the bread wheat accessions with high-quality sequenced genomes, except for the two landraces Chinese Spring and Norin61 (IWGSC 2018; Walkowiak et al. 2020; Sato et al. 2021; Aury et al. 2022). This observation highlights the need to include landraces in breeding programs to enlarge the genetic base of elite wheat varieties (Lopes et al. 2015; Marone et al. 2021). Future diversity studies of wheat should focus on landraces, in particular on those from regions with untapped genetic diversity, such as Turkey, Pakistan and Iran.

### Pathogen virulence characterization guides Pm gene discovery

To discover novel *R* genes against powdery mildew in the LandracePLUS panel, we used a set of ten *Bgt* isolates that showed highly diverse virulence patterns when tested on differential lines with single, known *Pm* genes. As we observed avirulence for many differential lines, using these isolates should reveal the presence of most *Pm* genes for which differential lines are available.

The tested wheat accessions were rarely resistant to more than three isolates, in line with characteristic *R* gene-based race-specific resistance (Flor 1971). This implies that the observed single resistance genes would be of limited agricultural use, depending on the isolates present in the corresponding wheat-growing area. To broaden the resistance spectrum and prevent fast evolution of pathogen virulence, such genes should be deployed in a suitable manner, e.g., by gene stacking, gene pyramiding or transgenic overexpression (Mundt 2018; Koller et al. 2019). On the other hand, the accessions of the LandracePLUS panel susceptible to all tested powdery mildew isolates can be assumed to lack any major resistance gene active at the seedling stage. Thus, observed resistance in adult plants in the field would likely be durable, making such accessions attractive donors of adult plant resistance.

Currently, for the 27 genes represented in the differential set of *Pm* lines used in this study, only eight of the corresponding avirulence genes are molecularly known (Bourras et al. 2015, 2019; Praz et al. 2017; Hewitt et al. 2021; Müller et al. 2022; Kloppe et al. 2023; Kunz et al. 2023). Avirulence gene sequence comparison in our ten powdery mildew isolates revealed the presence of recognized haplotypes of seven of the cloned Avrs. This knowledge guided us to determine whether a resistance-associated region in the LandracePLUS panel was derived from a known *Pm* gene, as we showed for the cloned gene *Pm2a*. Further, the information on resistance to *Bgt* isolates of distinct geographical origin can guide an informed deployment of accessions in the respective agricultural areas (Vleeshouwers and Oliver 2014; Müller et al. 2022). For example, our findings suggest further work on landraces resistant to CHN_46_30 for potential deployment in China because they must contain effective *R* genes against this isolate of Chinese origin.

### GWAS of the LandracePLUS panel detects known Pm genes and reveals six undescribed powdery mildew resistance-associated regions on chromosomes 1BL, 3BL, 5BL, 7BS and 7DL

GWAS for eight powdery mildew isolates on the LandracePLUS panel without subsetting revealed ten resistance-associated regions. Four of these were in genomic regions with previously described resistance genes. Of these, the peaks on chromosomes 2BL and 7AL overlapped with regions of genetically described, but not molecularly known *Pm* genes, while the peaks on chromosomes 1AS and 5DS were likely caused by the cloned genes *Pm3* and *Pm2*, respectively. While *Pm3* was known to be present in several LandracePLUS panel accessions (Bhullar et al. 2009, 2010a, b), we did not expect to find *Pm2* widely in the gene pool of landraces. *Pm2* was introduced into the breeding gene pool via the Russian cultivar Ulka (Pugsley and Carter 1953). It originated from the diploid wheat wild relative *Ae. tauschii* and has eight known haplotypes, of which only *Pm2a* was detected in hexaploid wheat (Manser et al. 2021). While *Pm2a* has been identified previously in six wheat landraces (Chen et al. 2019; Manser et al. 2021), we found its presence in 34, mostly Turkish landraces, suggesting Turkey as the geographical origin of the *Pm2a* resistance gene. While GWAS did not detect the cloned gene *Pm4* in the LandracePLUS panel, we discovered a high frequency of two non-functional *Pm4* alleles in Turkish landraces. Our findings suggest that these alleles originated in Turkey, fitting the origin of *Pm4* from tetraploid wheat, which also arose in Turkey (Özkan et al. 2002; Sánchez-Martín et al. 2021). Despite the narrow representation of *Pm4b* and *Pm4d* in landraces, a wide presence of these functional alleles compared to *Pm4f* and *Pm4g* has been described in elite germplasm (Sánchez-Martín et al. 2021). This suggests that the breeding process for *Pm4* mildew resistance was very effective.

We detected one region on chromosome 7DL not previously described as associated with mildew resistance. This region contained annotations for ten putative NLRs and one serine/threonine kinase. While NLRs are to date still the most common candidates for *Pm* genes, serine/threonine kinases have been described to play a role in defense response, including *Pto*, which confers resistance to bacterial speck disease in tomato (Martin et al. 1993; Loh and Martin 1995).

Using a *Pm2* covariate, we detected five additional undescribed resistance associations in the LandracePLUS panel. One of them was located on chromosome 7BS, containing candidate genes putatively encoding a C2H2-type zinc finger, a serine/threonine kinase and an F-box-like protein with LRR domains. The latter have been described to facilitate hypersensitive cell death response in tobacco and tomato (Burg et al. 2008) and shown to be involved in defense response to stripe rust in wheat (Yin et al. 2018). On the other hand, zinc fingers of the C2H2-type were linked to plant defense response (Kim et al. 2004; Tian et al. 2010; Yin et al. 2020; Sharma et al. 2021), where some cases have shown that the transcriptional repression activity of the zinc finger was the mechanism behind this association (Weigel et al. 2005; Uehara et al. 2005).

### Targeted GWAS subsets reveal 16 potentially novel resistance-associated loci

Utilizing targeted subsets, we discovered 16 most likely novel peaks on ten chromosomes (Table 2). GWAS is expected to be more powerful when conducted on large datasets where individuals are drawn randomly from the population (Uffelmann et al. 2021). However, important SNPs at a small regional scale might yet be diluted in species-wide panels and not detected by GWAS, which typically lacks the power to detect associations with rare alleles (Marees et al. 2018). Pending sufficient phenotypic and genetic variation, GWAS in local panels has proven very effective in *Arabidopsis thaliana* (Gloss et al. 2022). For this reason, resistance genes which may have been selected at small regional scales might be more efficiently detected in subsets of accessions from the same geographical origin.

Therefore, we focused on accessions originating from distinct countries that harbored novel genetic diversity, namely Pakistan, Iran and Turkey. This resulted in the detection of 16 additional loci where no *Pm* genes have been described. With a subset of accessions exclusively from Pakistan or Iran, we discovered a region on chromosome 6AS associated with mildew resistance. The region contains genes putatively encoding an NLR, a serine/threonine kinase and F-box-like proteins with LRR domains.

While we present novel regions and gene candidates, the molecular nature of the observed resistance must be confirmed in future studies, especially when considering that the reference genome Chinese Spring might lack (susceptible) alleles of the causal resistance genes. One approach would be the application of recent sequencing technologies, such as circular consensus sequencing (CCS) (Wenger et al. 2019). Such novel approaches have increased the feasibility of sequencing single donor accessions to assist in the cloning a gene of interest. For example, assembling the Kariega genome with CCS has demonstrated the usefulness of this approach in wheat and has led to the cloning of *Yr27* (Athiyannan et al. 2022a). Another option to identify a gene of interest in a specific genotype depends on the availability of a pangenome, which ideally would capture the entire gene repertoire of a species (Tettelin et al. 2005). High-quality sequencing efforts have recently resulted in 19 wheat genomes, of which 14 have reference genome quality (IWGSC 2018; Walkowiak et al. 2020; Sato et al. 2021; Aury et al. 2022; Athiyannan et al. 2022a; Kale et al. 2022). Genome analysis has revealed structural rearrangements, introgressions and differences in gene content (Walkowiak et al. 2020). A successful example of using this resource is the cloning of *Lr14a*, which was based on the reference genome ArinaLrFor (Kolodziej et al. 2021). However, despite the recent advances in pangenome projects, the close clustering of the high-quality sequenced genomes compared to the LandracePLUS panel suggests that the currently available pangenome includes only a fraction of the diversity present in landraces, including resistance loci. Thus, it is essential to include more diverse wheat accessions, specifically landraces, in future work to increase the extent of the pangenome, particularly for NLR loci, which are rarely present across wheat genotypes. We propose to assemble high-quality genomes of several landraces from Turkey, Pakistan and Iran to achieve such a goal. Furthermore, contrasting phenotypes for traits of interest should be included when setting up pangenome consortia. This diversified selection could provide a resource that guides various trait-genotype associations. Finally, we suggest choosing a donor accession with confirmed resistance for each peak and sequencing the resistance-associated genome using CCS. This will reveal which gene candidates are present in the specific accessions and guide their molecular cloning and validation, e.g., via virus-induced gene silencing (Cakir et al. 2010).

The subset-based association analysis done in this work of the genetically diverse LandracePLUS panel challenged with ten *Bgt* isolates unraveled 22 potentially novel powdery mildew resistance genes. Therefore, this study can be used as an example for future work on similar collections in search of other traits of interest. Once a diversity panel is assembled, instead of focusing on single pathogen races, phenotyping with diverse isolates from geographically defined agricultural regions followed by subset-based analyses for the origin of accessions would reveal more resistance loci compared to studies done with single pathogen isolates on entire collections.

### Supplementary Information

Below is the link to the electronic supplementary material.

Supplementary file1 (PDF 9320 KB)

Supplementary file2 (XLSX 785 KB)
Table S1. Information on accessions of the LandracePlus panel. Alleles with asterisks are non-functional. Groups are based on the hierarchical clustering of the LandracePLUS panel, including high-quality sequenced genomes.
Table S2. Differential lines used for determining powdery mildew isolate virulence spectra and the respective Pm genes they contain. NILs had been backcrossed multiple times with susceptible accessions “Federation” or “Chancellor”, depicted by /x*Accession, where x is the number of backcrosses to the designated accession (McIntosh et al. 2013).
Table S3. Sampling information of the ten Bgt isolates.
Table S4. Molecularly known Avr and Svr haplotypes of the ten powdery mildew isolates used for phenotyping. H0 = published avirulent haplotype, H1 = published virulent haplotype, H2 = unpublished haplotype. Based on the phenotype of the differential lines containing cognate Pm genes, we propose that H2 of AvrPm1a and AvrPm3a/f are virulent, while H2 of AvrPm3d is proposed to be avirulent. H2 of AvrPm17 is likely virulent due to the resemblance of H1, with just one additional amino acid change. The resistant phenotype of the differential line containing Pm17 is suggested to be due to an additional Pm gene in Amigo. Amino acid (AA) changes are given based on H0 and depicted with original AA, position, new AA.
Table S5. Heritability of phenotypes for all ten powdery mildew isolates based on a linear mixed model.
Table S6. Correlation of genetic relatedness and phenotypes for the ten *Bgt* isolates.
Table S7. Gene candidates and annotations in Chinese Spring for regions associated to powdery mildew resistance. Highlighted genes are the best candidates based on their annotation.
Table S8. List of all significantly associated SNPs, their position, MAF, effect size beta and *p*-values for the LandracePLUS panel excluding subsets.
Table S9. List of all significantly associated SNPs, their position, MAF, effect size beta and *p*-values for the subsets of the LandracePLUS panel
Table S10. Raw phenotype data of the LandracePLUS panel infected with ten powdery mildew isolates. Plates refer to the individual petri dish the replicate was in and Rounds to a batch that was infected at the same time.

Supplementary file3 (XLSX 303 KB) Alleles in the 20 most modern cultivars of the LandracePLUS panel for each potentially novel resistance-associated region. Resistance-associated alleles and accessions with more than 50% of these alleles and a resistance phenotype toward the peak-producing isolate are highlighted in bold. In addition, an overview of SNPs for all detected resistance-associated loci and the 755 wheat accessions with passport data and disease reaction is provided.

Supplementary file4 (XLSX 282) Candidate genes in chromosome-scale-assembled wheat varieties ArinaLrFor, Fielder, Jagger, Julius, Kariega, Lancer, Landmark, Mace, SY Mattis, Norin61, Renan and Stanley based on syntenic analysis to regions associated to powdery mildew resistance in CS.

Supplementary file Data S1. Raw genotyping data of the LandracePLUS panel with the TaBW35K SNP array. Empty cells are labeled “vide”, and wheat accession IDs are extended by “_cell identifier”.

Supplementary file Data S2. Sample statistics of the raw genotyping data of the LandracePLUS panel. Total numbers are labeled “nb” and percentage “pc”.

Supplementary file Data S3. Marker statistics of the raw genotyping data of the LandracePLUS panel. Total numbers are labeled “nb” and percentage “pc”.

## Data Availability

The mildew isolate CHE_19004 whole-genome sequence data are available at NCBI’s Short Read Archive (SRA) under the accession code BioProject PRJNA945619. The nine isolate sequences from earlier work (Sotiropoulos et al. 2022) are available at NCBI under PRJNA625429 and SRP062198. The genotyping data of landraces from the study of Balfourier and colleagues (Balfourier et al. 2019) can be accessed at https://urgi.versailles.inra.fr/download/wheat/genotyping/Balfourier_et_al_Wheat_Phylogeography_DataS2.zip. All other data needed to evaluate the conclusions in this study are included in the article and its supplementary information files. All *Blumeria graminis* f.sp. *tritici* isolates used in this study are kept alive in the Department of Plant and Microbial Biology of the University of Zurich and are available upon request.
