# Supplementary material for: A diverse panel of 755 bread wheat accessions harbors untapped genetic diversity in landraces and reveals novel genetic regions conferring powdery mildew resistance
Source: Theor Appl Genet. 2024 Mar 27;137(4):88. doi: 10.1007/s00122-024-04582-4 (PMC10965746; doi:10.1007/s00122-024-04582-4)
Supplement: Supplementary file 1 — Supplementary file1 (PDF 9320 KB) [file 122_2024_4582_MOESM1_ESM.pdf]

Supplementary to

A diverse panel of 755 bread wheat accessions harbors untapped genetic diversity in landraces and reveals novel genetic regions conferring powdery mildew resistance

Rebecca Leber[1], Matthias Heuberger[1], Victoria Widrig[1,2], Esther Jung[1], Etienne Paux[3,4], Beat Keller[1]* & Javier Sánchez-Martín[1,2]*

[1] University of Zurich, Department of Plant and Microbial Biology, Zollikerstrasse 107, 8008 Zurich, Switzerland

[2] Department of Microbiology and Genetics, Spanish-Portuguese Agricultural Research Center (CIALE), University of Salamanca, 37007 Salamanca, Spain

[3] Université Clermont Auvergne, INRAE, GDEC, 63000 Clermont-Ferrand, France

[4] VetAgro Sup Campus agronomique, 63370 Lempdes, France

* Corresponding authors

E-mail: j.sanchezmartin@usal.es, bkeller@botinst.uzh.ch

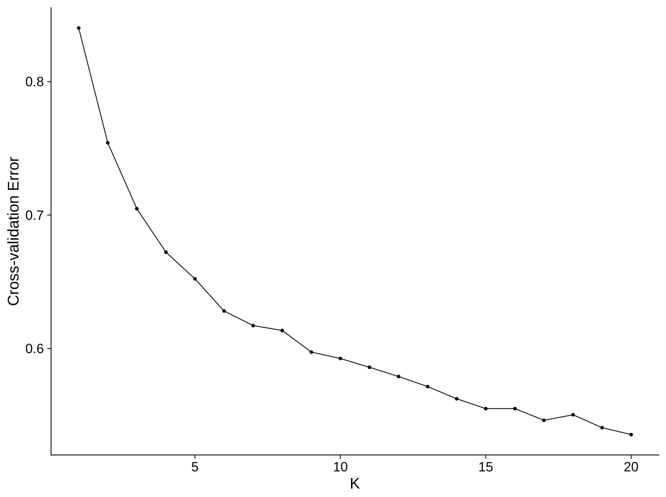

Figure S1. Cross-validation error of Admixture kinship analysis for K=2 to K=20 for 29,965 SNPs

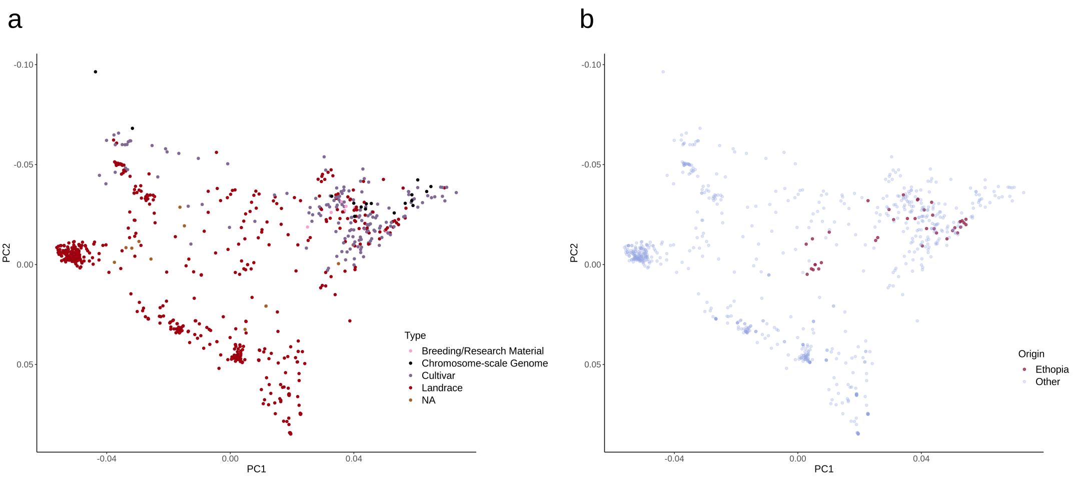

Figure S2. PCAs from 27,337 SNPs of the LandracePLUS panel including high-quality sequenced genomes with PC1 = 8.4% and PC2 = 5.1%
a Types of wheat accessions are highlighted in different colors. **b** Ethiopian landraces are highlighted by color in comparison to accessions of another origin

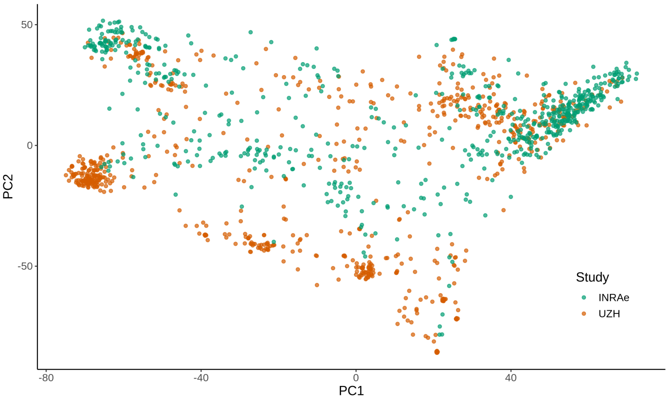

Figure S3. Genetic diversity of the LandracePLUS panel compared to 632 landraces from an INRAe study that represent the original pool of worldwide hexaploid diversity (Balfourier et al. 2019)

PCA from the filtered 29,965 polymorphic SNPs that were present in SNP arrays of both studies. PC1 = 8.9%, PC2 = 4.0%. Accessions from the LandracePLUS panel are shown in orange, while INRAe landraces are colored in green

a

b

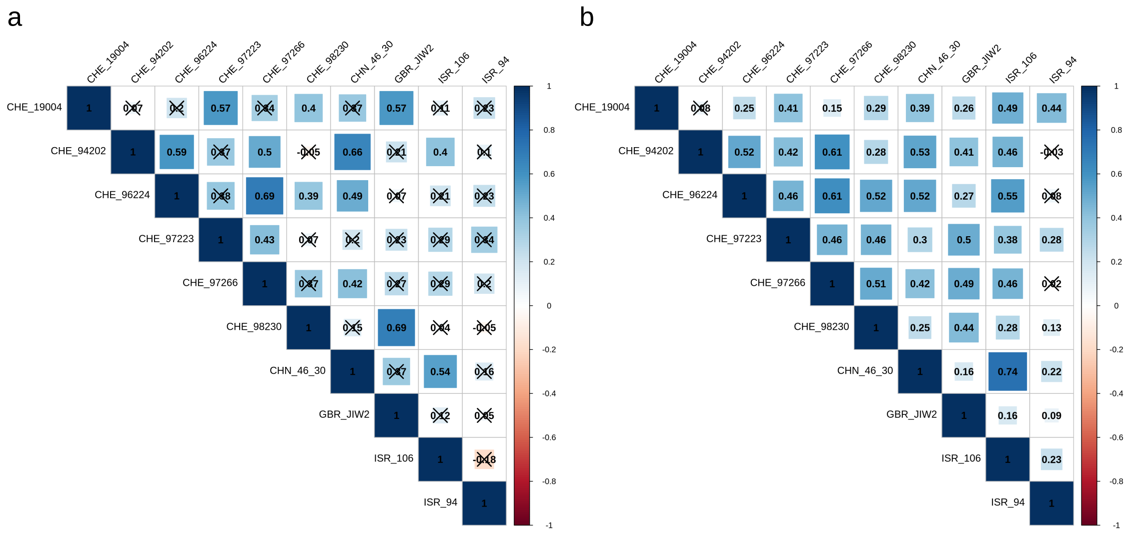

Figure S4. Correlation matrix showing Pearson's correlation of virulence between the ten isolates used for phenotyping

a Correlation coefficients based on phenotypic variation of the differential lines. **b** Correlation coefficients based on phenotypic variation in the LandracePLUS panel. Correlation coefficients with corresponding p-values below the significance 0.05 are crossed out

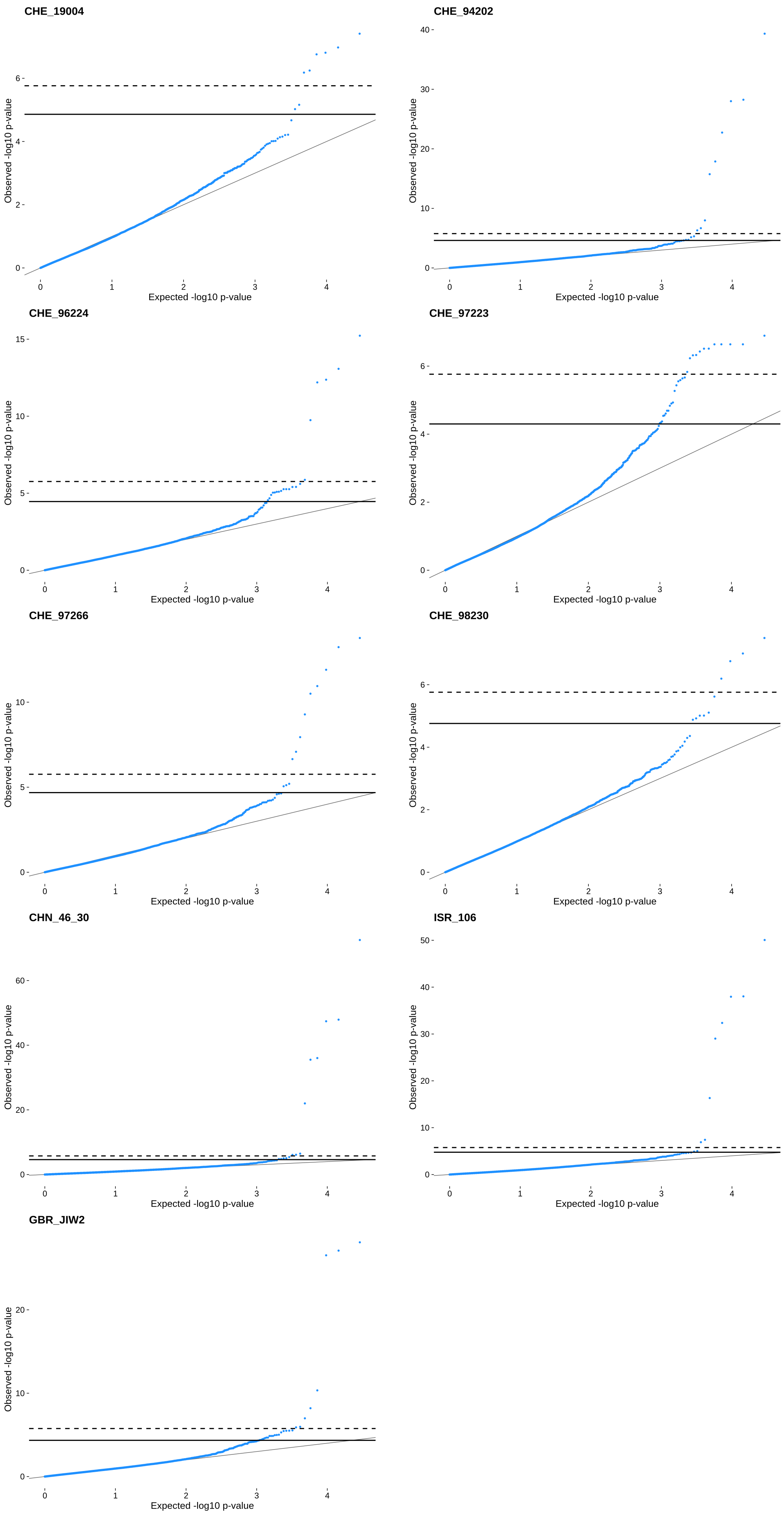

Figure S5. QQ Plots for GWAS with nine powdery mildew isolates and the LandracePLUS panel
Isolate names are depicted in the top-left corner. Solid lines represent the threshold for False discovery rate (FDR) and dashed lines for Bonferroni correction

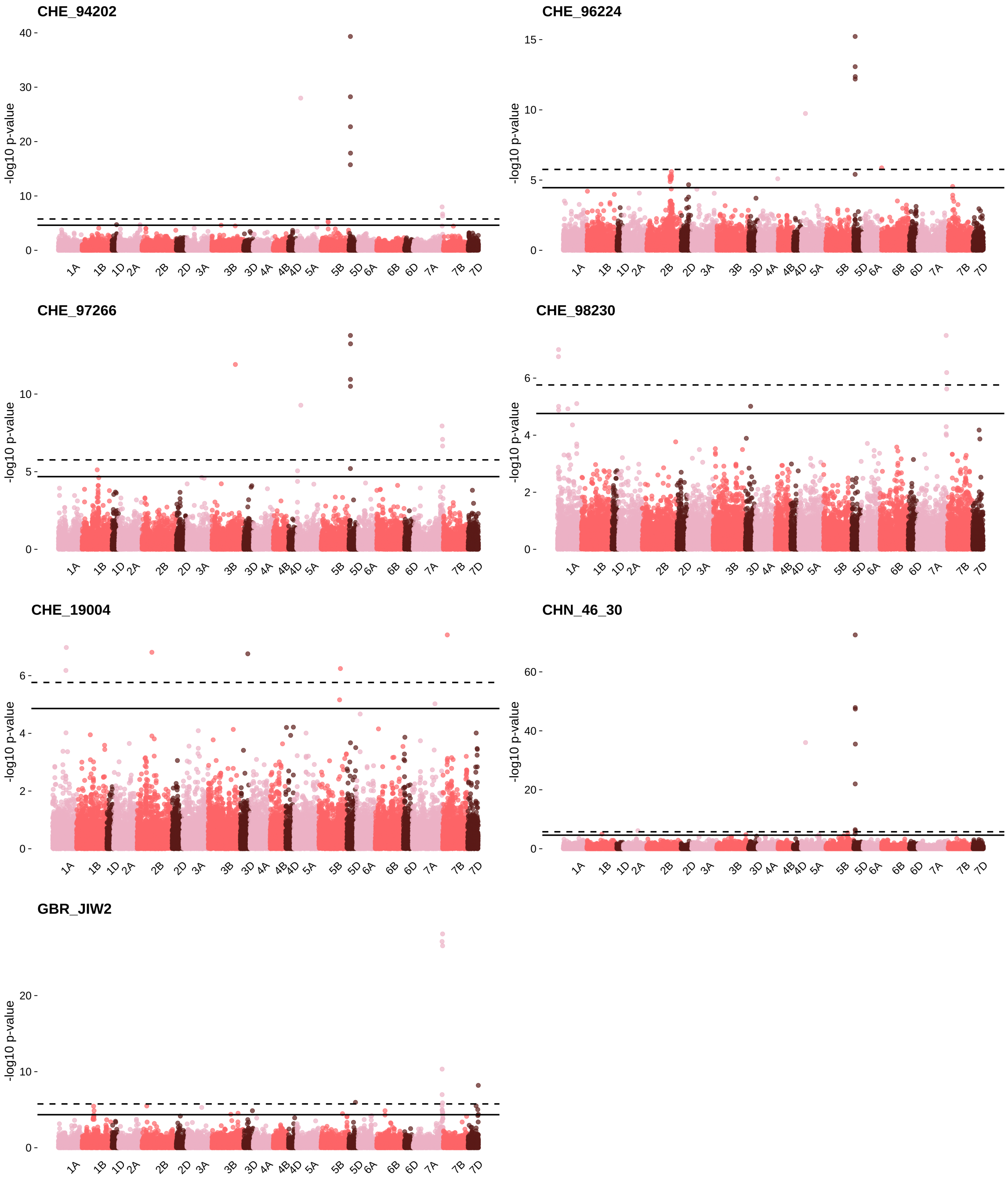

Figure S6. Manhattan Plots for GWAS with the seven powdery mildew isolates and the LandracePLUS panel that showed a good fit in the QQ plots for the univariate linear mixed model
Isolate names are depicted in the top-left corner. Solid lines represent the threshold for False discovery rate (FDR) and dashed lines for Bonferroni correction

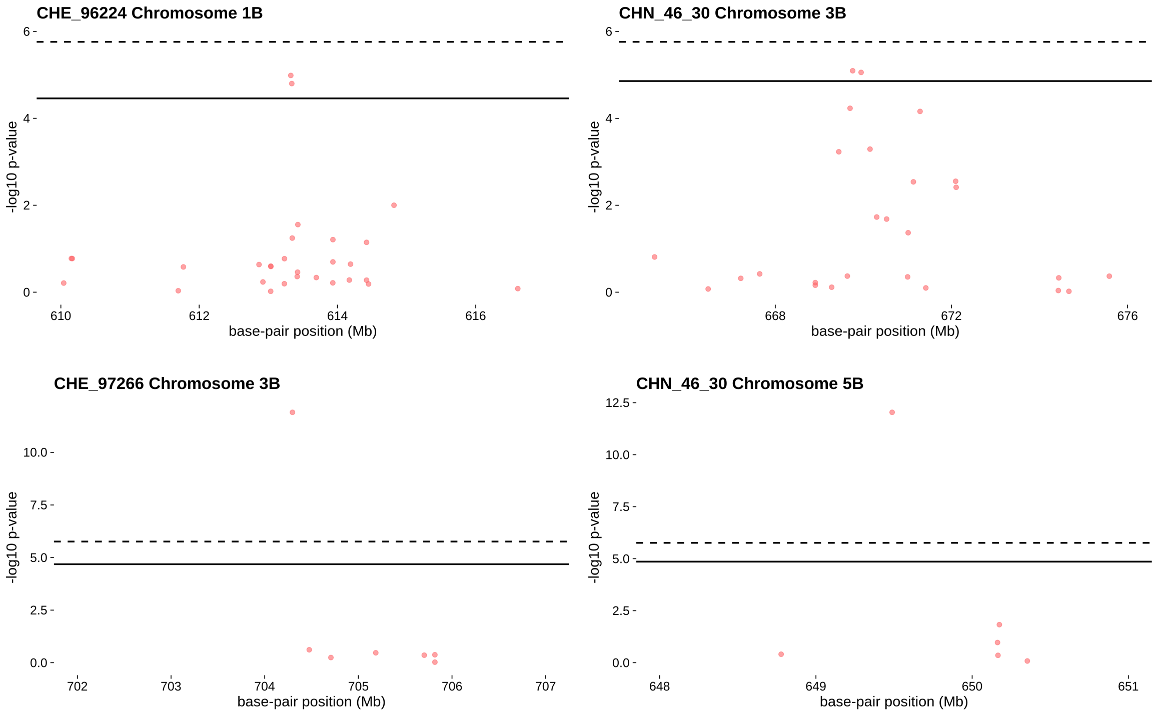

Figure S7. Manhattan Plots for GWAS of the LandracePLUS panel using a *Pm2* covariate and the isolates that contain *AvrPm2*

Isolate and chromosomes are depicted in the top-left corner. Solid lines represent the threshold for False discovery rate (FDR) and dashed lines for Bonferroni correction

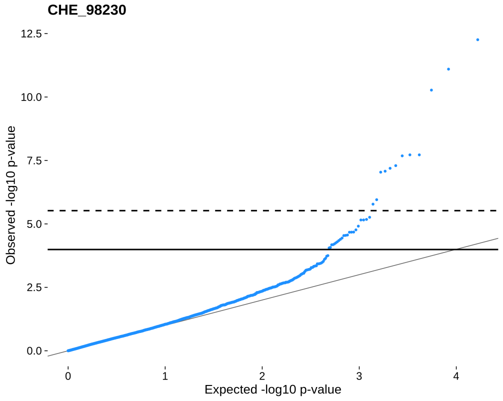

Figure S8. QQ Plot for GWAS with powdery mildew isolate CHE_98230 and landraces from Pakistan and Iran

Isolate names are depicted in the top-left corner. Solid lines represent the threshold for False discovery rate (FDR) and dashed lines for Bonferroni correction

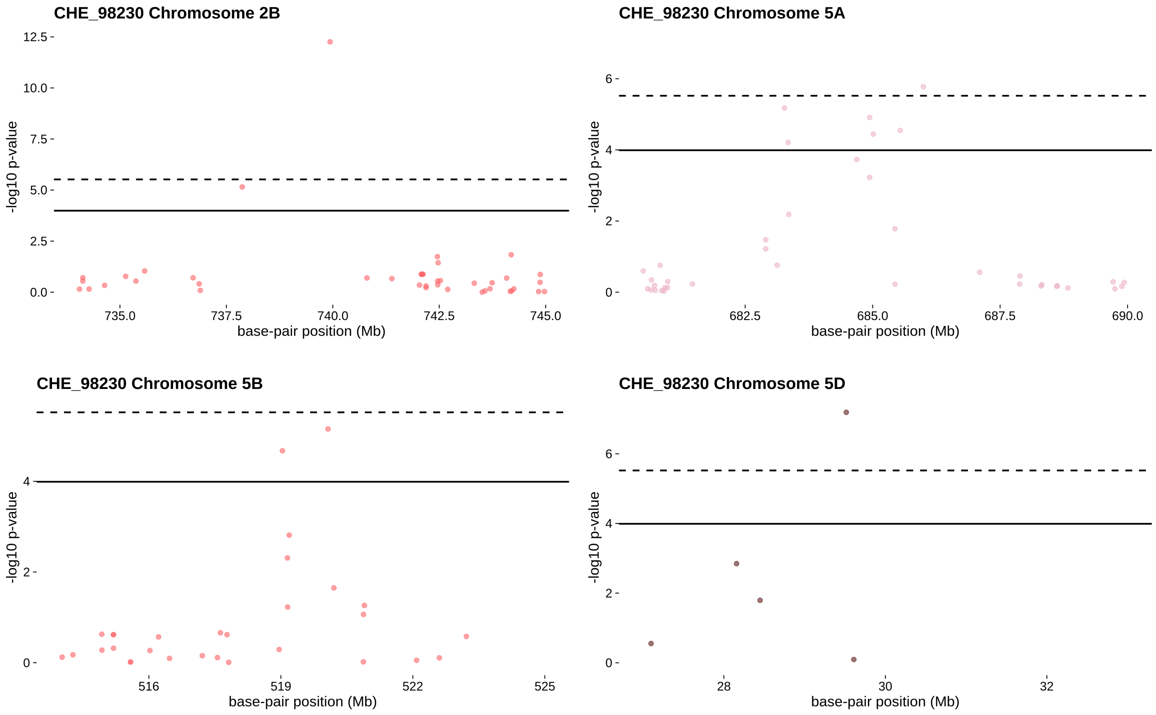

Figure S9. Manhattan Plots of CHE_98230 resistance-associated loci of accessions from Pakistan and Iran Isolate and chromosomes are depicted in the top-left corner. Solid lines represent the threshold for False discovery rate (FDR) and dashed lines for Bonferroni correction

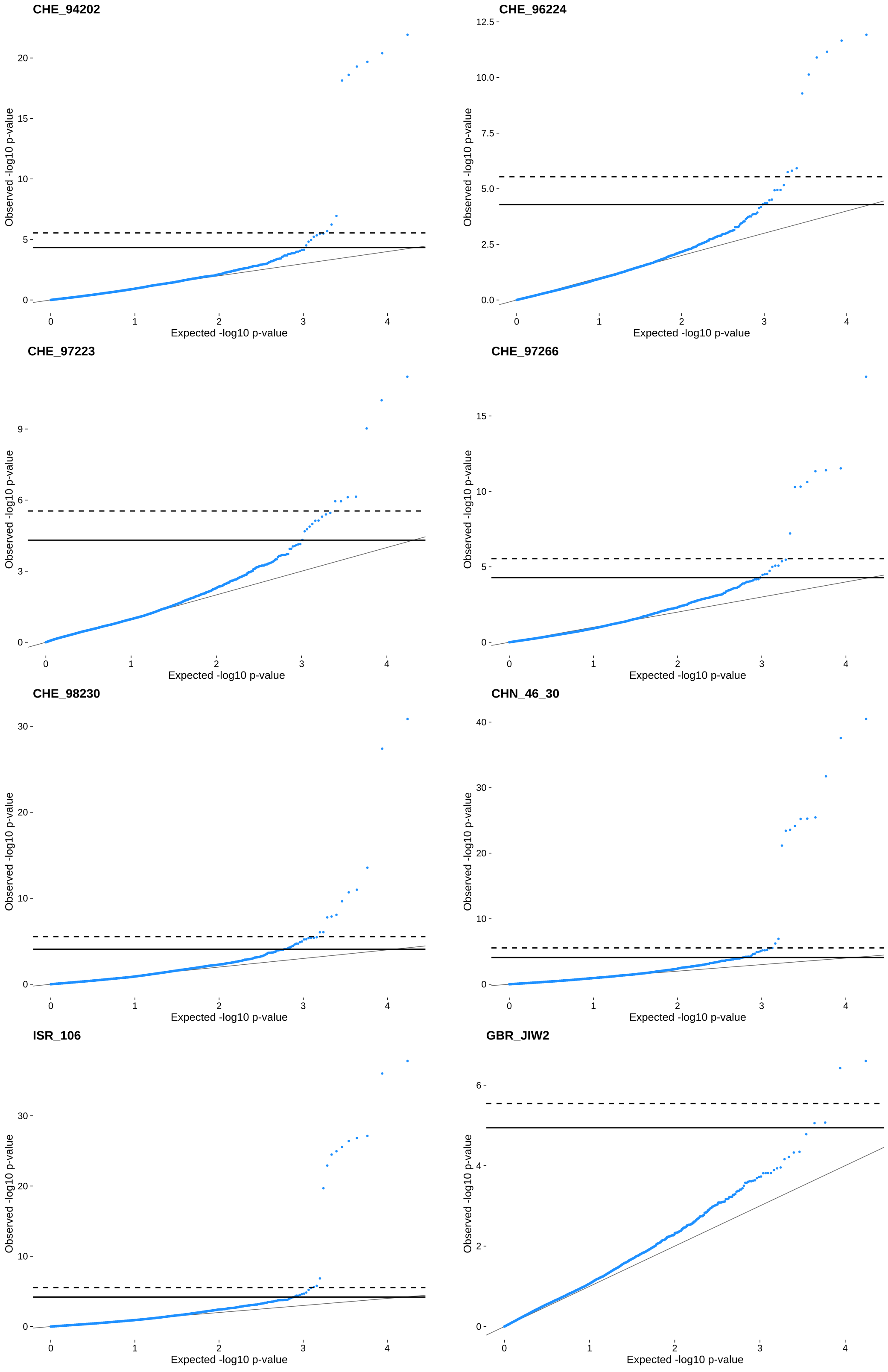

Figure S10. QQ Plots for GWAS with eight powdery mildew isolates and landraces from Turkey
Isolate names are depicted in the top-left corner. Solid lines represent the threshold for False discovery rate (FDR) and dashed lines for Bonferroni correction

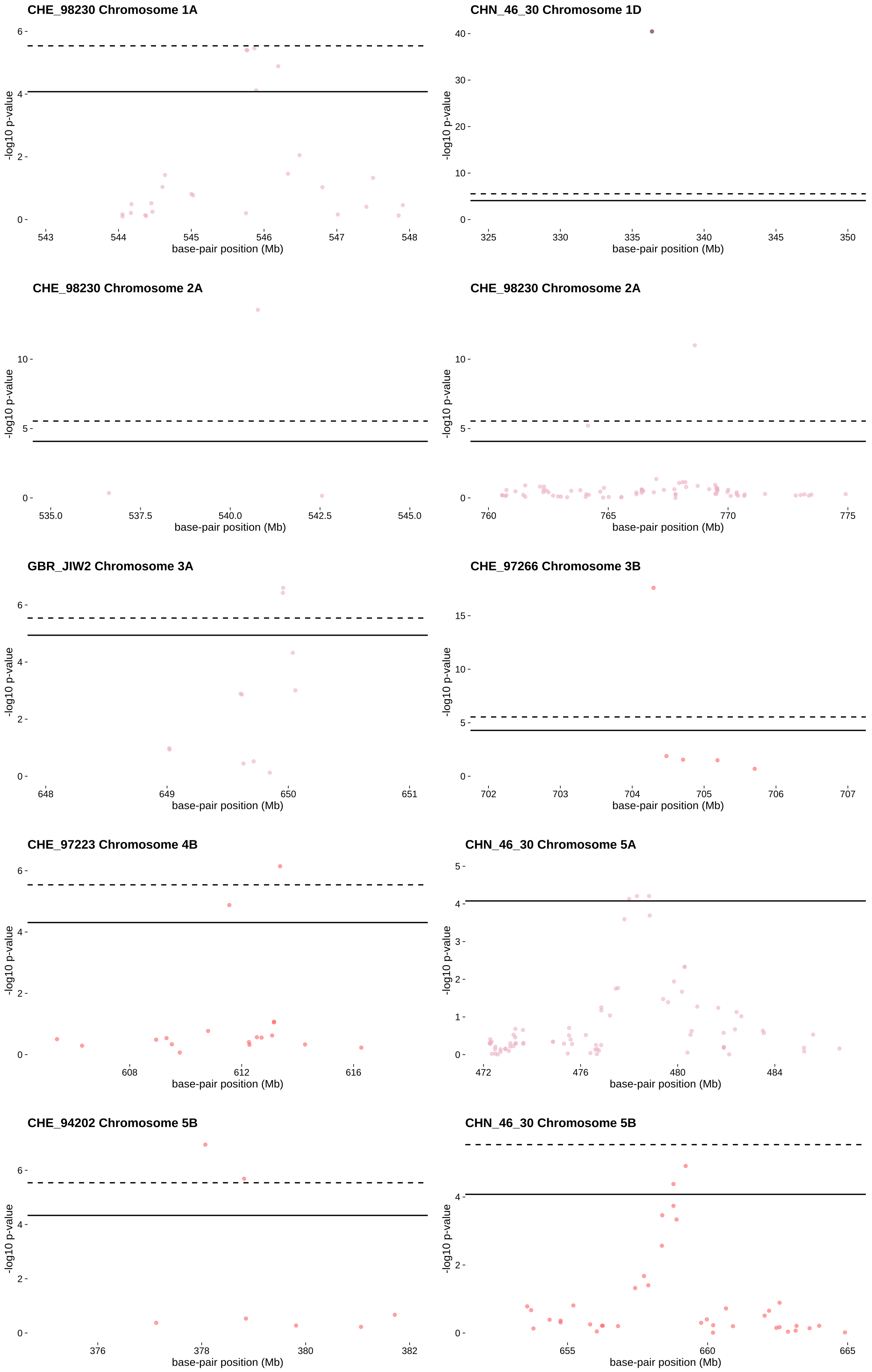
CHE_98230 Chromosome 1A
-log10 p-value
base-pair position (Mb)
543 544 545 546 547 548
0 2 4 6
CHN_46_30 Chromosome 1D
-log10 p-value
base-pair position (Mb)
325 330 335 340 345 350
0 10 20 30 40
CHE_98230 Chromosome 2A
-log10 p-value
base-pair position (Mb)
535.0 537.5 540.0 542.5 545.0
0 5 10
CHE_98230 Chromosome 2A
-log10 p-value
base-pair position (Mb)
760 765 770 775
0 5 10
GBR_JIW2 Chromosome 3A
-log10 p-value
base-pair position (Mb)
648 649 650 651
0 2 4 6
CHE_97266 Chromosome 3B
-log10 p-value
base-pair position (Mb)
702 703 704 705 706 707
0 5 10 15
CHE_97223 Chromosome 4B
-log10 p-value
base-pair position (Mb)
608 612 616
0 2 4 6
CHN_46_30 Chromosome 5A
-log10 p-value
base-pair position (Mb)
472 476 480 484
0 1 2 3 4 5
CHE_94202 Chromosome 5B
-log10 p-value
base-pair position (Mb)
376 378 380 382
0 2 4 6
CHN_46_30 Chromosome 5B
-log10 p-value
base-pair position (Mb)
655 660 665
0 2 4

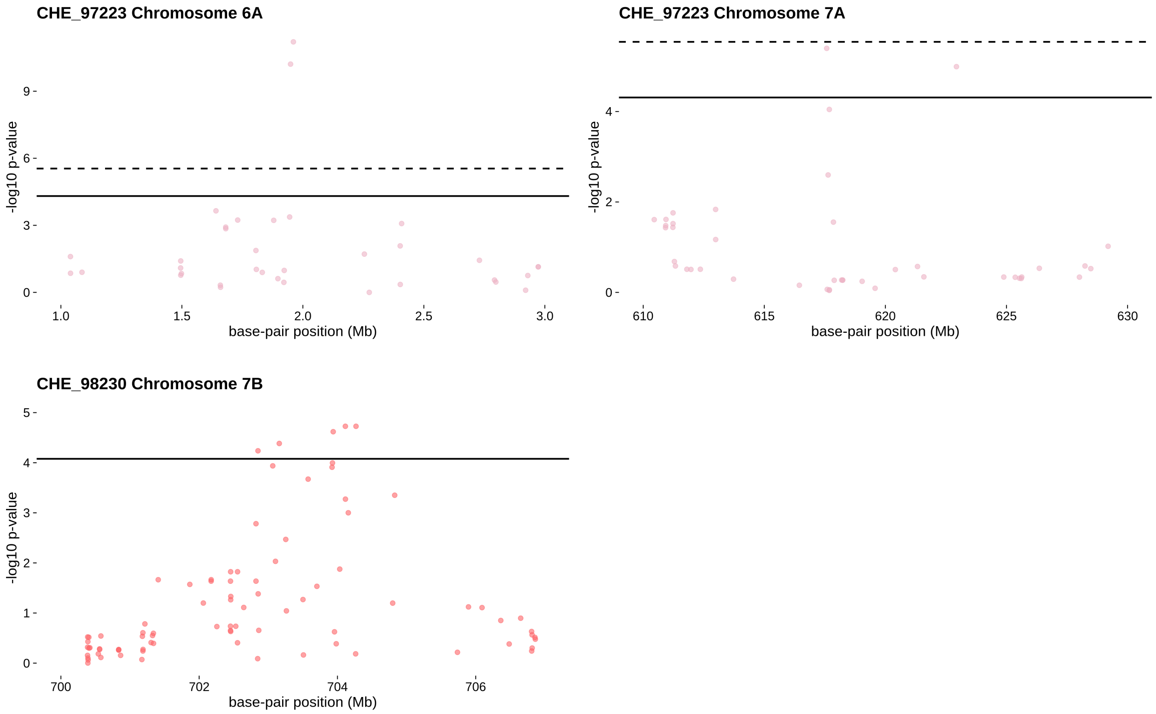

Figure S11. Manhattan Plots of resistance-associated loci of Turkish accessions
Isolates and chromosomes are depicted in the top-left corner. Solid lines represent the threshold for False discovery rate (FDR) and dashed lines for Bonferroni correction. Associations that occurred for several isolates are represented by one isolate only

References

Balfourier F et al. (2019) Worldwide phylogeography and history of wheat genetic diversity. *Sci Adv* 5.
